# Aging‐Related Muscle *Bmal1* Decline Contributes to Bone Loss in Mice via Enhancing IL‐1α–Mediated Osteoclastogenesis

**DOI:** 10.1111/acel.70582

**Published:** 2026-06-08

**Authors:** Kai Huang, Jun Qian, Yike Wang, Feng Zhang, Youjia Xu, Qiaocheng Zhai

**Affiliations:** ^1^ Orthopaedic Institute Wuxi Ninth People's Hospital Affiliated to Soochow University Wuxi Jiangsu China; ^2^ Division of Orthopaedics The Quzhou Affiliated Hospital of Wenzhou Medical University, Quzhou People's Hospital Quzhou Zhejiang China; ^3^ Department of Orthopaedics The Second Affiliated Hospital of Soochow University Suzhou Jiangsu China

**Keywords:** circadian clock, Hmox1, osteoclast, osteoporosis, time‐restricted feeding

## Abstract

Osteoporosis, a common metabolic bone disorder linked to aging, is often accompanied by muscle degeneration. Muscle *Bmal1* disruption in mice has been shown to affect various tissues, including the kidney, lung, and bone, indicating that the muscle molecular clock may influence the physiological homeostasis of multiple organs through systemic circulation. Despite this, the role of the muscle clock in age‐related osteoporosis remains unclear. In aged mice, we observed a disruption in the circadian interaction between muscle and bone. Furthermore, both *Bmal1* expression within muscle fibers and total BMAL1 protein levels in muscle tissue were significantly reduced. Using skeletal muscle‐specific *Bmal1* knockout mice, we observed osteoporosis‐related phenotypes, including decreased bone mass and disrupted trabecular microarchitecture, along with disrupted diurnal expression of inflammatory cytokines. Mechanistically, we revealed that muscle *Bmal1* deficiency impairs the rhythmic expression of the *Hmox1* and induces the upregulation of IL‐1α in muscle cells. The elevated circulating IL‐1α promotes osteoclast differentiation, ultimately reducing bone mass. Importantly, the osteoporosis‐related phenotype resulting from muscle *Bmal1* knockout or aging was alleviated by nighttime time‐restricted feeding (TRF), which reestablished a feeding‐driven diurnal variation in *Hmox1* expression within the muscle and reduced serum IL‐1α levels. This study provides new insights into the pathogenesis of osteoporosis in the aging population. Furthermore, it suggests that TRF may offer a promising therapeutic strategy for treating osteoporosis in the elderly.

## Graphical abstract

Aging‐related decline of muscle *Bmal1* drives bone loss in mice by disrupting *Hmox1* and increasing IL‐1α, which promotes osteoclast activity. Time‐restricted feeding reestablishes diurnal *Hmox1* expression, reduces IL‐1α, and alleviates osteoporosis, offering a potential therapeutic strategy.

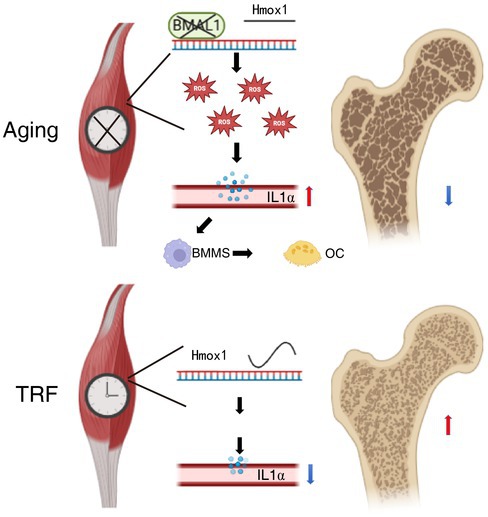

## 1 Introduction

The biological clock is an intrinsic molecular mechanism that enables the body to synchronize with the light–dark cycle. The BMAL1‐CLOCK heterodimer binds to the E‐Box element to regulate the expression of core clock genes, such as *Per* and *Cry*, and other clock‐controlled genes. The PER and CRY proteins undergo phosphorylation in the cytoplasm, enter the nucleus, and suppress the transcriptional activity of BMAL1 and CLOCK. Furthermore, REV‐ERBs and RORs inhibit and activate the transcription of BMAL1 and CLOCK, respectively. This process is known as the transcription‐translation feedback loop (Takahashi 2017). Disruptions in biological rhythms are strongly linked to aging. At the individual level, a 16‐year study involving 1000 elderly participants revealed a significant decline in the strength and stability of sleep‐activity rhythms with age (Cai et al. 2023). At the tissue level, RNA transcriptome analysis in mice revealed that aging leads to a reduction in the number of rhythmic genes across various tissues and organs, accompanied by substantial changes in the phase of circadian gene expression (Wolff et al. 2023). Additionally, mice deficient in the core circadian clock gene *Bmal1* exhibit signs of premature aging, including sarcopenia, cataracts, subcutaneous fat loss, and organ atrophy, highlighting the circadian clock's role in aging regulation (Kondratov et al. 2006).

Osteoporosis, a prevalent chronic disease in the elderly, is characterized by reduced bone mass, increased fragility, and a higher risk of osteoporotic fractures (Watts and Manson 2017). At the cellular level, bone metabolism homeostasis depends on the coordinated activity of osteoblasts, osteocytes, and osteoclasts. An imbalance between osteoblast‐mediated bone formation and osteoclast‐driven bone resorption contributes to bone metabolic disorders (Raisz 2005). Research indicates that biological rhythms play a crucial role in regulating bone homeostasis. Shift workers with disrupted circadian rhythms and individuals with sleep disorders face a significantly higher risk of osteoporosis than those with stable sleep–wake cycles (Feskanich et al. 2009; Quevedo and Zuniga 2010; Sasaki et al. 2016). Similarly, mice lacking core circadian clock genes exhibit impaired metabolic balance between osteoblasts and osteoclasts, leading to altered bone metabolism‐related phenotypes (Goncalves and Meng 2019). Moreover, knocking out *Bmal1* in osteoblasts and bone marrow mesenchymal stem cells (BMSCs) reduces bone mass by enhancing osteoclast activity (Takarada et al. 2017; Tsang et al. 2019). BMAL1 also directly regulates *Nfatc1* transcription, promoting osteoclast differentiation, while its deletion in osteoclasts increases bone mass (Xu et al. 2016). These findings highlight the essential role of intrinsic *Bmal1* in bone tissue for maintaining balanced bone metabolism.

Skeletal muscle is the largest organ involved in energy metabolism and endocrine function in the human body. It is closely linked to bone tissue and can influence bone metabolism through mechanical stress and the secretion of cytokines (Brotto and Bonewald 2015). A recent study has shown that exosomes derived from skeletal muscle can prevent disuse osteoporosis caused by muscle atrophy by promoting osteogenic differentiation of BMSCs (Ma et al. 2023). This finding suggests that exosomes may also regulate bone metabolism as key muscle‐bone coupling factors. Growing interest has focused on the role of muscle molecular clocks in regulating the physiological functions of other organs. For instance, a study has shown that knocking out *Bmal1* in muscle tissue increases nonrapid eye movement sleep in mice and alleviates the negative effects of sleep deprivation (Ehlen et al. 2017). Additionally, muscle *Bmal1* deficiency elevates the expression of IL‐6 and TIMP‐1 inflammatory factors in the kidney and alters the immune cell composition in lung tissue (Crislip et al. 2022), indicating that *Bmal1*‐deficient muscle tissue may disrupt the physiological homeostasis of other organs through cytokine secretion. Further investigation revealed that the specific knockout of *Bmal1* in skeletal muscle caused thickening of the distal tibia, increased calcification of the calcaneal tendon, and reduced ankle cartilage in mice (Schroder et al. 2015). These findings suggest that impaired muscle‐bone circadian coupling may contribute to bone metabolic disorders.

In conclusion, we hypothesize that muscle *Bmal1* plays a critical role in regulating age‐related osteoporosis. To investigate this, we first conducted time‐series RNA‐seq analyses to investigate potential alterations in rhythmic genes and circadian interactions between muscle and bone tissues in aged mice. We also specifically analyzed alterations in *Bmal1* expression within aged muscle and generated skeletal muscle‐specific *Bmal1* knockout mice using CKMM‐cre mice to explore the role and regulatory mechanisms of the skeletal muscle biological clock in osteoporosis.

## 2 Results

### 2.1 Age‐Related Remodeling of Muscle‐Bone Crosstalk in Mice

**FIGURE 1:**
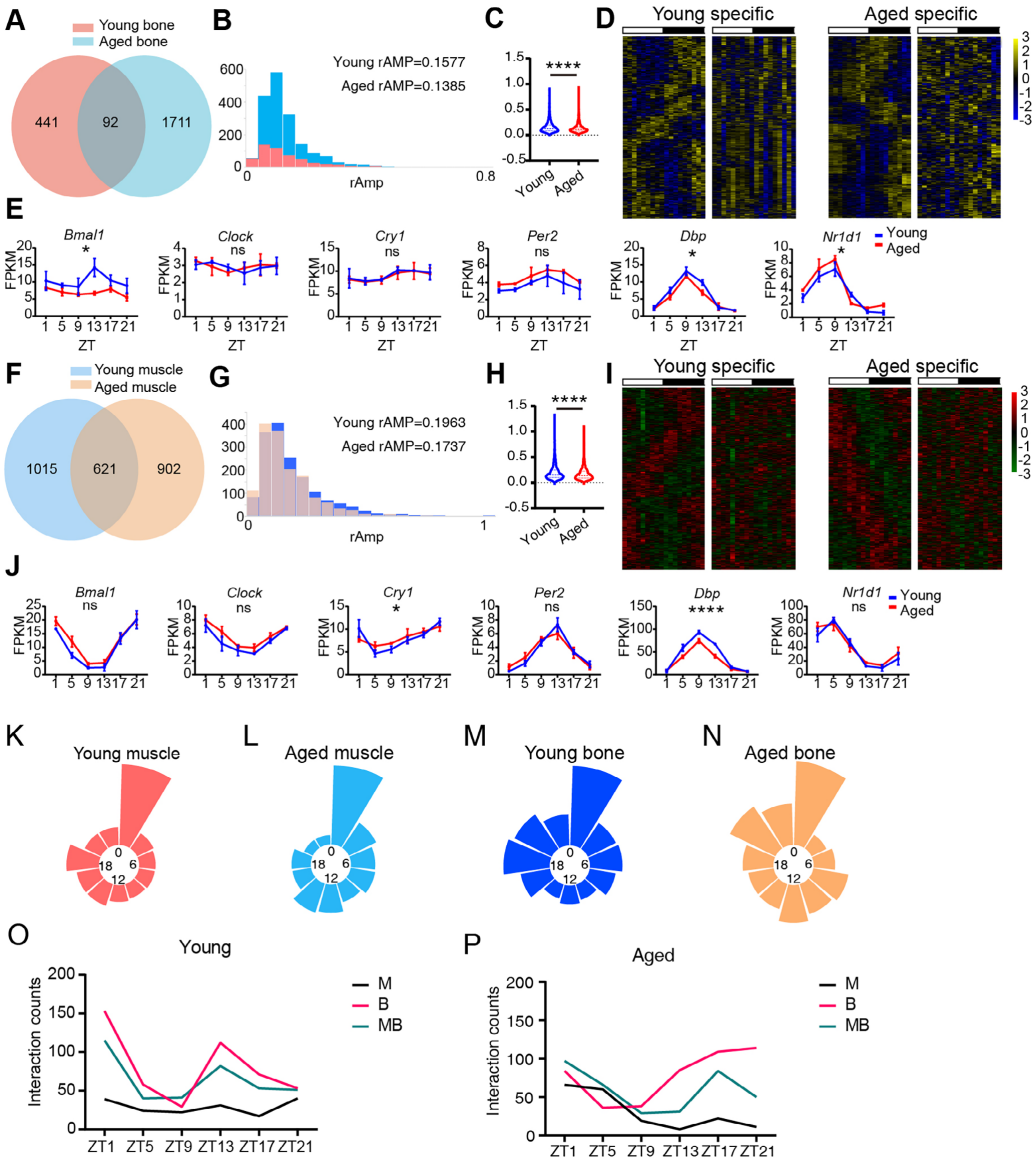
Circadian remodeling of muscle‐bone tissue crosstalk during aging. (A, F) Venn diagrams depicting rhythmic genes in the tibia and gastrocnemius muscle of young (2‐month‐old) and aged (18‐month‐old) mice across six circadian time points (ZT1, 5, 9, 13, 17, 21; *n* = 3 mice per time point per age group, where ZT indicates Zeitgeber Time, hours after lights‐on). (B, C, G, H) Amplitude distribution of rhythmic genes in the tibia and gastrocnemius muscle of young and aged mice. (D, I) Heatmaps displaying genes exhibiting age‐specific rhythmicity in the tibia and gastrocnemius muscle (Young‐specific: Rhythmic only in young mice; Aged‐specific: Rhythmic only in aged mice). (E, J) Expression profiles (FPKM, Fragments Per Kilobase of transcript per Million mapped reads) of core clock genes in the tibia and gastrocnemius muscle of young and aged mice. Data are presented as mean ± SD. Differences in gene expression were analyzed using two‐way ANOVA. (K–N) Phase distribution of rhythmic genes in the gastrocnemius muscle and tibia of young and aged mice. (O, P) Temporal profiling of significantly rhythmic receptor‐ligand interactions at distinct time points: (M) Within muscle, (B) Within bone tissue, and (MB) Between muscle and bone tissues in young and aged mice.

To investigate age‐related changes in circadian rhythms within murine muscle and bone, gastrocnemius muscle and tibia tissues were harvested from 2‐month‐old (young) and 18‐month‐old (aged) mice at six circadian time points (ZT1, ZT5, ZT9, ZT13, ZT17, ZT21, where ZT indicates Zeitgeber Time, hours after lights‐on) following 1 week of adaptation to a light–dark (LD) cycle. RNA‐seq analysis coupled with MetaCycle methodology revealed significant tissue‐specific remodeling of the circadian transcriptome during aging. In bone tissue, the number of rhythmic genes was greater in aged mice compared to young controls. Only 92 rhythmic genes were common between young and aged groups (Figure 1A), with 441 genes losing rhythmicity in aged bone while 1711 genes acquired novel rhythmicity (Figure 1A,D). Furthermore, the amplitude of rhythmic genes was significantly lower in aged bone (Figure 1B,C). Two‐way ANOVA revealed that the expression of core clock genes, specifically *Bmal1*, *Dbp*, and *Nr1d1*, was significantly altered in aged mice compared to young controls, with a notable reduction in the amplitude of *Bmal1* observed in aged bone (Figure 1E). Conversely, in skeletal muscle, the total number of rhythmic genes showed a modest decrease in aged mice. A core set of 621 rhythmic genes was common to both groups (Figure 1F), while rhythmicity was lost for 1015 genes in aged muscle and 902 genes gained novel rhythmicity (Figure 1F,I). Aged muscle also exhibited a significant reduction in the amplitude of rhythmic genes (Figure 1G,H). The rhythmic expression profiles of core clock genes remained largely unchanged in aged muscle, with the exception of *Cry1* and *Dbp* (Figure 1J). Besides, micro‐CT and molecular analyses established that 18‐month‐old aged mice develop distinct osteoporotic bone changes and skeletal muscle senescence (Figure S1). Additionally, while 18‐month‐old mice exhibited a significant reduction in cortical thickness, we observed a concurrent increase in cortical bone mineral density and cortical area (Figure S1G–I). This combination of decreased thickness and increased total area suggests an age‐related structural remodeling of the cortical shell, where compensatory periosteal expansion occurs alongside endosteal bone loss (Ferguson et al. 2003; Shim et al. 2022). Collectively, these results indicate a profound reorganization of the circadian transcriptome in both muscle and bone tissues of aged mice.

To investigate potential age‐related alterations in circadian crosstalk between muscle and bone, we first analyzed the phase distribution of rhythmic gene expression. In both tissues, the highest density of rhythmic genes peaked during the ZT1‐3 in both young and aged mice (Figure 1K,L). Notably, based on the continuous phase values calculated by MetaCycle, young skeletal muscle exhibited a secondary high‐density distribution bin of rhythmic genes peaking between ZT17 and ZT19 (Figure 1K). In aged muscle, this phase distribution shifted, with the secondary concentration of rhythmic phases occurring earlier, between ZT13 and ZT15 (Figure 1L). In bone tissue, rhythmic genes were predominantly concentrated in the ZT1‐3 window in young mice. However, the phase distribution across other time intervals was also altered in aged bone (Figure 1M,N). These results indicate phase remodeling of the rhythmic transcriptome in both tissues during aging. To assess the functional implications of this phase shift on intratissue signaling and muscle‐bone crosstalk, we employed CellphoneDB to analyze temporal changes in receptor‐ligand pairs. Within young bone tissue, intratissue interactions peaked at ZT1 and ZT13, whereas in aged bone, the peak interaction number occurred at ZT21 (Figure 1O,P). Intramuscular interactions in young mice lacked a significant temporal peak, but aged muscle exhibited a distinct peak at ZT1 (Figure 1O,P). Furthermore, analysis of muscle‐bone intertissue receptor‐ligand pairs revealed the first peak at ZT1 in both groups. However, the second peak shifted from ZT13 in young mice to ZT17 in aged mice (Figure 1O,P). To specifically explore the potential impact of muscle‐derived secreted protein on bone, we analyzed muscle ligand‐bone receptor pairs across all time points. This revealed a comprehensive remodeling in aged mice, encompassing changes in the repertoire of interacting pairs, their interaction strength, and the phase of peak interaction (Figure S2A,B), underscoring a profound reorganization of muscle‐to‐bone signaling dynamics with age.

These findings suggest that the circadian transcriptome undergoes reprogramming in both muscle and bone tissues of aged mice. Furthermore, the rhythmic signaling from muscle to bone is significantly altered during aging. This disruption in circadian coupling between muscle and bone may underlie the age‐related bone loss observed in these animals.

### 2.2 Skeletal Muscle Cell‐Specific *Bmal1* Knockout Induces Osteopenia in Male Mice

**FIGURE 2:**
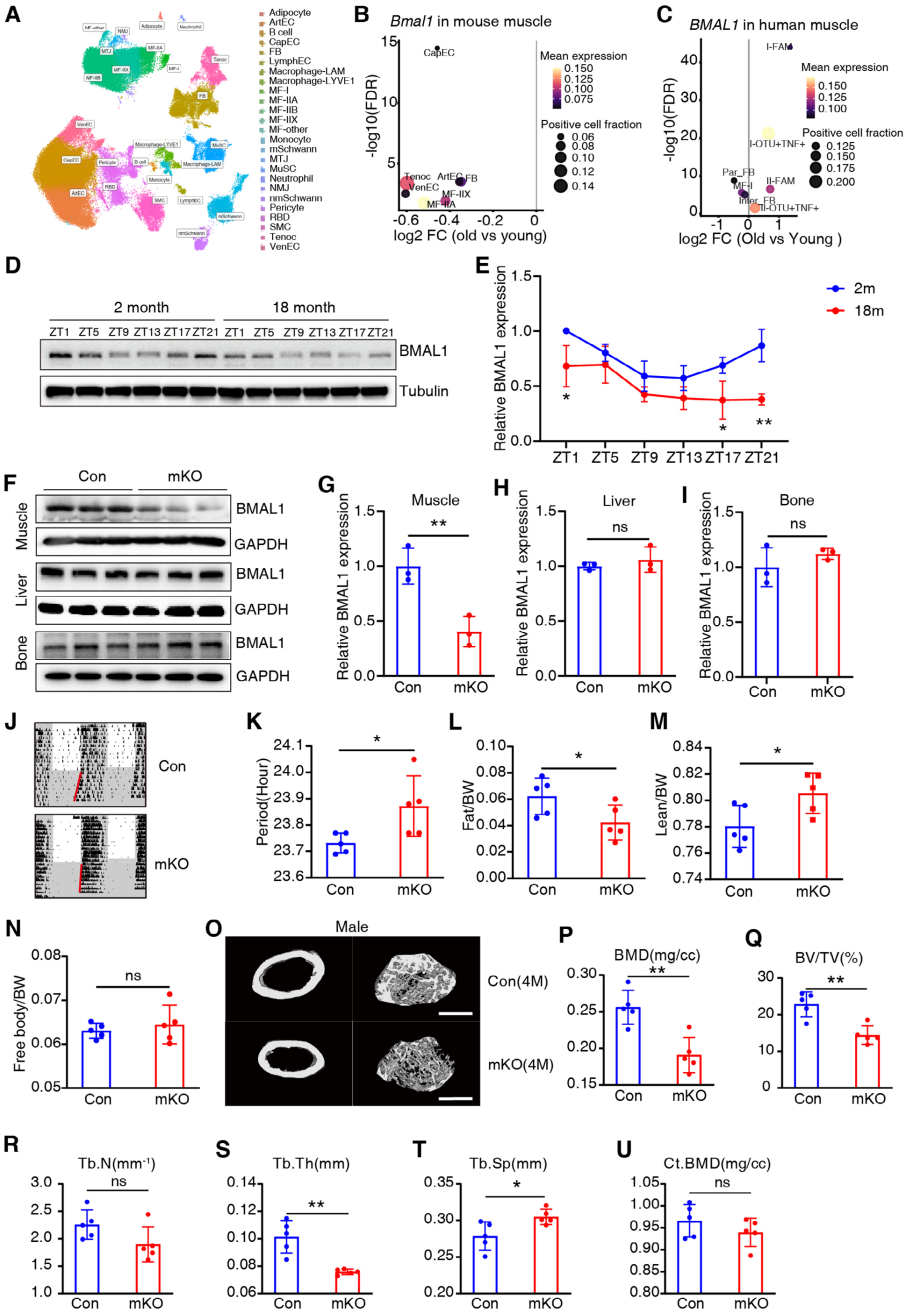
Skeletal muscle‐specific *Bmal1* deficiency reduces bone mass. (A) Single‐cell UMAP projections illustrating *Bmal1* expression in skeletal muscle from mice. (B) Cell types exhibiting differential *Bmal1* expression in the skeletal muscle of aged (18‐month‐old) versus young (3‐month‐old) mice (*p* < 0.05, |Fold change| > 0). (C) Cell types exhibiting differential *BMAL1* expression in the skeletal muscle of aged (50–75 years) versus young (15–40 years) human subjects (*p* < 0.05, |Fold change| > 0, mean expression > 0.05). (D) Temporal expression profile of BMAL1 protein in the gastrocnemius muscle of 2‐month‐old and 18‐month‐old mice across six ZT points (ZT 1, ZT 5, ZT 9, ZT 13, ZT 17, ZT 21). (E) Quantification of BMAL1 expression levels at different ZT points (*n* = 3 per time point). (F–I) Western blots and quantification of BMAL1 protein expression in gastrocnemius muscle, liver and tibia of skeletal muscle‐specific *Bmal1* knockout (mKO) mice and their littermate controls (*n* = 3). (J) Representative double‐plotted actograms depicting wheel‐running activity under 12‐h light: 12‐h dark cycles (LD; days 1–10) and constant darkness (DD; days 11–20) for mKO and control mice (*n* = 5). Gray‐shaded areas indicate dark phases. (K) Quantification of free‐running period (tau, *τ*) in DD for mKO and control mice (*n* = 5). (L–N) Body composition analysis: Ratios of fat mass, lean mass, and free fat mass to total body weight in mKO and control mice (*n* = 5). (O) Representative 3D micro‐CT reconstructions of cortical and trabecular bone in the distal femur of male mKO and control mice (scale bar: 1 mm). (P–T) Quantitative micro‐CT analysis of trabecular bone parameters in the distal femur: (P) Bone mineral density (BMD), (Q) Bone volume fraction (BV/TV), (R) Trabecular number (Tb.N), (S) Trabecular thickness (Tb.Th), and (T) Trabecular separation (Tb.Sp) (*n* = 5). (U) Cortical bone mineral density (Ct.BMD) at the femoral midshaft in male mKO and control mice (*n* = 5). Data are presented as mean ± SD. *p < 0.05, **p < 0.01, ns, not significant.

*Bmal1* serves as a core transcription factor within the molecular circadian clock. Because bulk RNA‐seq represents a heterogeneous mixture of cells that can mask myofiber‐specific changes, we analyzed single‐cell RNA sequencing data from both human and mouse samples to examine alterations in *Bmal1* expression during skeletal muscle aging at a higher cellular resolution (Kedlian et al. 2024). Compared to young mice, aged mice exhibited a significant reduction in *Bmal1* expression within type II myofibers (Figure 2A,B). Furthermore, among all differentially expressed cell types, the mean expression level of *Bmal1* was highest in myofibers (Figure 2B). Similarly, *Bmal1* expression was significantly lower in the type I myofibers of aged human muscle compared to young human muscle (Figures 2C and S3A). Consistent with these findings, analysis of an independent murine scRNA‐seq dataset (Aging Atlas Consortium 2021) confirmed a significant reduction of *Bmal1* expression in 16‐month‐old mice compared to 2‐month‐old mice across multiple cell populations, including type IIB fast‐twitch fibers, fibro‐adipogenic progenitors/fibroblasts, pericytes, endothelial cells, smooth muscle cells, myotendinous junction cell, neuromuscular junction precursor cell, macrophages, and tendons (Figure S3B). Notably, this age‐related decrease was most pronounced in type IIB fast‐twitch fibers (Figure S3B). To validate these transcriptomic changes at the protein level, we collected gastrocnemius muscle samples from 2‐month‐old young mice and 18‐month‐old aged mice at 4‐h intervals. Western blot analysis showed a marked decrease in BMAL1 expression in the gastrocnemius muscle of aged mice at ZT1, ZT17 and ZT21 compared to young mice (Figure 2D,E). Taken together, these results resolve an important discrepancy: while our initial bulk RNA‐seq analysis showed no significant change in overall *Bmal1* mRNA levels—likely due to the masking effect of nonmyogenic cell populations within the highly heterogeneous whole‐muscle tissue—high‐resolution single‐cell analysis and protein quantification confirm that *Bmal1* expression is indeed significantly and specifically reduced within individual myofibers of aged skeletal muscle.

To assess the potential impact of muscle circadian clock disruption on bone mass, we generated skeletal muscle‐specific *Bmal1* knockout (mKO) mice by mating *Bmal1* flox^+/+^ mice with CKMM‐cre^+/−^ *Bmal1* flox^+/+^ mice to mimic *Bmal1* downregulation on aged myofibers. WB analysis confirmed that BMAL1 expression was significantly reduced in the gastrocnemius muscle, while BMAL1 levels in the liver and bone remained unchanged (Figure 2F–I). We next assessed the wheel running behavior of the mice and found that the intrinsic circadian period of muscle *Bmal1* knockout mice was significantly prolonged compared to control mice (Figure 2J,K). Since the running rhythm of mice is regulated by the core circadian clock in the suprachiasmatic nucleus (SCN) and its downstream neural signaling pathways, this result suggests that *Bmal1*‐deficient muscles may secrete factors that influence the core clock. Body composition analysis revealed that knockout mice had significantly reduced body fat content and increased lean mass, with no significant difference in body fluid content (Figure 2L–2N). Furthermore, the weights and muscle fiber cross‐sectional areas of both the gastrocnemius and quadriceps were significantly greater in knockout mice compared to the control group, accompanied by a significant downregulation of myostatin (*Mstn*) expression (Figure S4). These findings indicate an overall increase in muscle mass in the knockout mice, which is consistent with previous report (Liu et al. 2025). These findings indicate that skeletal muscle‐specific *Bmal1* knockout may influence other organs via secreted proteins in the circulatory system. Additionally, the downregulation of *Bmal1* in skeletal muscle cells does not appear to be the primary cause of muscle loss associated with aging.

Next, we collected femoral tissues from 4‐month‐old male mice and performed micro‐CT analysis. In male mice, the results showed significant reductions in cancellous bone density, BV/TV, and trabecular thickness in knockout mice compared to control mice. Additionally, the trabecular spacing was significantly increased in knockout mice (Figure 2O–T). The knockout mice exhibited no significant differences in cortical bone density compared with control femur (Figure 2U). In 10‐month‐old male mice, we observed significant reductions in cancellous bone density, BV/TV, trabecular number, and trabecular thickness in knockout mice compared to control mice, accompanied by a significant increase in trabecular separation, indicating further deterioration of the trabecular microarchitecture (Figure S5). These results indicate that skeletal muscle cell‐specific *Bmal1* knockout leads to bone loss and trabecular bone microstructural deterioration in male mice. Thus, reduced expression of *Bmal1* in skeletal muscle cells may contribute to the development of age‐related osteoporosis.

### 2.3 Skeletal Muscle *Bmal1* Deficiency Promotes Osteoclast Differentiation

**FIGURE 3:**
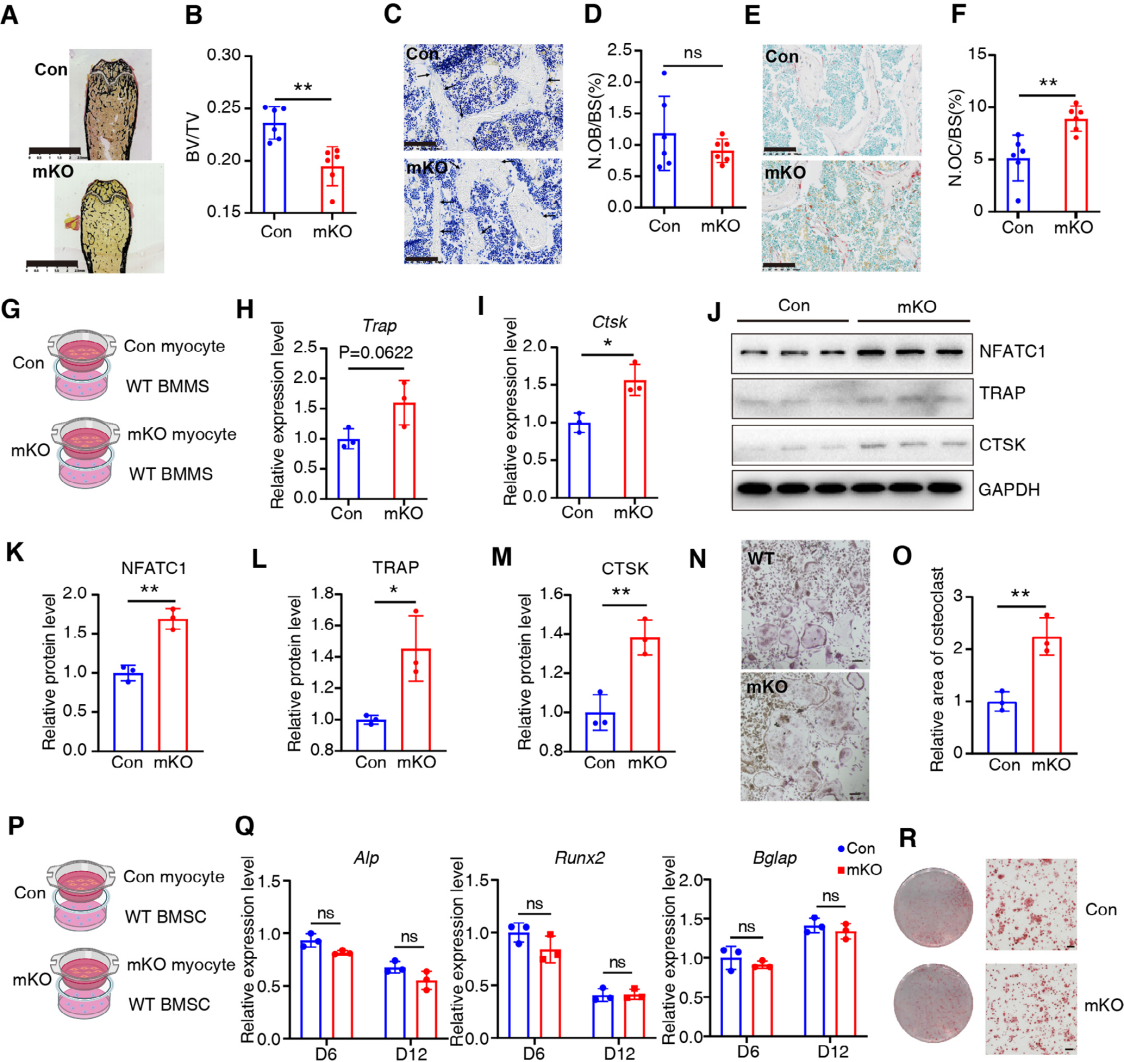
Skeletal muscle *Bmal1* deficiency promotes osteoclast differentiation. (A) Representative von Kossa staining of femoral sections from mKO mice and littermate controls, indicating mineralized bone matrix (scale bar: 2.5 mm). (B) Quantification of mineralized bone area fraction from von Kossa staining (*n* = 6). (C) Representative toluidine blue staining of femoral trabecular bone sections from mKO and control mice (black arrows indicate osteoblasts, scale bar: 100 μm). (D) Quantification of osteoblast number per bone surface (N.Ob/BS) from toluidine blue‐stained sections (*n* = 6). (E) Representative tartrate‐resistant acid phosphatase (TRAP) staining of femoral sections from mKO and control mice, identifying osteoclasts (scale bar: 100 μm). (F) Quantification of osteoclast number per bone surface (N.Oc/BS) from TRAP‐stained sections (*n* = 6). (G) Schematic diagram of the coculture system utilizing primary myocytes and wild‐type BMMs. (H, I) Relative mRNA expression levels of osteoclast markers *Trap* (H) and *Ctsk* (I) in BMMs cocultured for 7 days with primary myocytes isolated from mKO or control mice, under osteoclastogenic conditions (*n* = 3). (J–M) Representative western blots (J) and quantification of NFATC1 (K), TRAP (L) and CTSK (M) protein expression in cocultured BMMs on day 7 of osteoclast differentiation (*n* = 3). (N) Representative TRAP staining of cocultured BMMs on day 7 of osteoclast differentiation (scale bar: 100 μm). (O) Quantification of TRAP‐positive multinucleated osteoclasts formed in coculture (*n* = 3). (P) Schematic diagram of the coculture system utilizing primary myocytes and BMSCs. (Q) Relative mRNA expression levels of osteoblast markers *Alp*, *Runx2* and *Bglap* in BMSCs cocultured with primary myocytes from mKO or control mice, measured on day 6 and day 12 of osteogenic differentiation (*n* = 3). (R) Effects of primary myocytes from control and *Bmal1* knockout mice on the mineralization of BMSCs (Scale bar: 200 μm). Data are presented as mean ± SD. *p < 0.05, **p < 0.01, ns, not significant.

The balance between bone formation, regulated by osteoblasts, and bone resorption, controlled by osteoclasts, is crucial for maintaining bone homeostasis. Von Kossa staining of femur sections revealed that femoral mineralization was significantly reduced in skeletal muscle cell‐specific *Bmal1* knockout mice compared to control mice (Figure 3A,B). To investigate the cause of the bone mass decrease due to *Bmal1* knockout in skeletal muscle cells, we performed toluidine blue and tartrate‐resistant acid phosphatase (TRAP) staining. We observed no significant difference in osteoblast number between knockout and control mice (Figure 3C,D), but the number of osteoclasts was significantly higher in the knockout mice (Figure 3E,F). These findings suggest that *Bmal1* knockout in skeletal muscle cells may lead to bone mass loss by promoting osteoclast differentiation. To test this hypothesis, we isolated primary skeletal muscle cells from control and *Bmal1* knockout mice. We then cocultured these primary skeletal muscle cells with wild‐type (WT) primary bone marrow‐derived macrophages (BMMs) (Figure 3G). The results showed that *Bmal1* knockout skeletal muscle cells promoted the expression of TRAP and CTSK at both the mRNA and protein levels in WT BMMs (Figure 3H–M). Furthermore, TRAP staining revealed that *Bmal1* knockout skeletal muscle cells enhanced osteoclast differentiation of WT BMMs compared to control skeletal muscle cells (Figure 3N,O). In addition, by coculture of *Bmal1* knockout skeletal muscle cells with WT BMSCs, we evaluated both the expression of osteogenic differentiation markers (*Alp*, *Runx2*, and *Bglap*) and the ultimate bone‐forming functional activity via mineralization assays. We found that there was no significant difference between the mKO and control groups (Figure 3P–R). The observed time‐dependent decline in *Alp* and *Runx2* expression from day 6 to day 12 reflects the normal physiological transition of osteoblasts from the differentiation phase into the mature mineralization phase. Importantly, *Bglap*, a late‐stage osteoblast marker, was increased at day 12 compared with day 6 under both control and mKO coculture conditions. This finding supports progression toward late‐stage osteoblast maturation rather than a nonspecific suppression of osteogenic gene expression, without any observable cytotoxic effects in the coculture system.

These findings suggest that *Bmal1* knockout in skeletal muscle cells may regulate osteoclast differentiation through a secretory pathway.

### 2.4 *Bmal1* Knockout in Skeletal Muscle Cells Disrupts Rhythmic Expression of Antioxidant‐Related Genes

**FIGURE 4:**
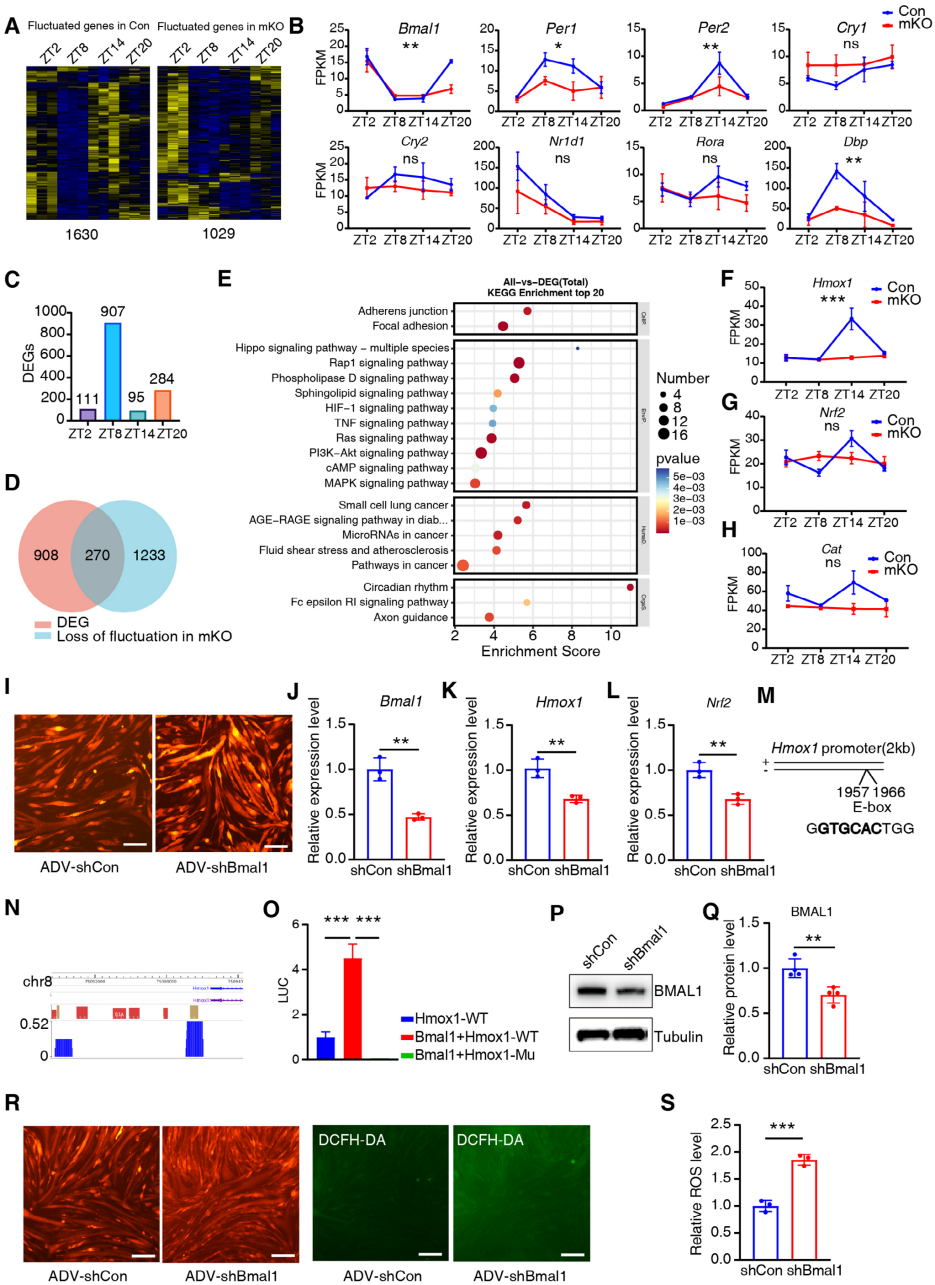
BMAL1 regulates *Hmox1* transcription and ROS levels in myotubes. (A) Heatmap of rhythmic genes identified in the gastrocnemius muscle of mKO mice and littermate controls across circadian time points. (B) FPKM of core clock genes in the gastrocnemius muscle of mKO and control mice. (C) Number of DEGs between mKO and control mice at ZT2, ZT8, ZT14, and ZT20 (*p* < 0.05 and fold changes ≥ 2). (D) Venn diagram illustrating the overlap between DEGs (from any ZT point) and genes exhibiting loss of rhythmicity in mKO muscle. (E) KEGG pathway enrichment analysis of 270 genes identified as both differentially expressed and losing rhythmicity in mKO muscle. (F–H) FPKM expression profiles of antioxidant genes *Hmox1* (F), *Nrf2* (G), and *Cat* (H) in the gastrocnemius muscle of mKO and control mice. (I) Representative fluorescence micrographs demonstrating efficient transduction of C2C12 myotubes with adenovirus (ADV) constructs (scale bar: 200 μm). (J–L) Relative mRNA expression levels of *Bmal1* (J), *Hmox1* (K), and *Nrf2* (L) in C2C12 myotubes following *Bmal1* knockdown using ADV‐shRNA, as quantified by RT‐PCR (*n* = 3). (M) Schematic diagram of predicted E‐box elements within the mouse *Hmox1* promoter region. (N) Publicly available ChIP‐seq data (Cistrome Database IDs: 73295 heart) demonstrating BMAL1 binding signals within the *Hmox1* promoter region in multiple mouse tissues. (O) Dual‐luciferase reporter assay assessing transcriptional regulation of the *Hmox1* promoter by BMAL1 (*n* = 3). 293 T cells were cotransfected with expression vectors for BMAL1 or control, along with either a wild‐type (*Hmox1*‐WT) or mutant (*Hmox1*‐Mut) *Hmox1* promoter‐driven luciferase reporter construct. (P) Western blot analysis confirming BMAL1 knockdown efficiency in ADV‐shBmal1‐transduced C2C12 myotubes. (Q) Quantification of BMAL1 protein levels (*n* = 3). (R) Representative fluorescence micrographs showing intracellular reactive oxygen species (ROS) levels detected by DCFH‐DA staining in control and *Bmal1* knockdown (ADV‐shBmal1) C2C12 myotubes (scale bar: 200 μm). Red fluorescence indicates viral transduction (mCherry or similar marker), green fluorescence indicates ROS signal. (S) Quantification of intracellular ROS levels in control and *Bmal1* knockdown C2C12 myotubes (*n* = 3). Data are presented as mean ± SD. *p < 0.05, **p < 0.01, ***p < 0.001, ns, not significant.

To investigate the mechanism by which skeletal muscle *Bmal1* regulates osteoclast differentiation, we collected gastrocnemius muscles from mice every 6 h under a 12:12 light–dark cycle. Gene expression was measured at ZT2, ZT8, ZT14, and ZT20 using RNA sequencing. To account for the limited phase estimation precision associated with a 4‐time‐point design, we applied a stringent minimum amplitude threshold (Amp > 0.1) alongside a *p* < 0.05 cutoff during MetaCycle analysis. This strategy mitigates potential false positives from lower‐frequency sampling and ensures the identification of robust circadian disruptions. In the gastrocnemius muscles of control mice, we identified a total of 1630 rhythmic genes, whereas 1029 rhythmic genes were identified in *Bmal1* knockout mice (Figure 4A). We further examined the expression of core clock genes in the gastrocnemius muscle. In *Bmal1* knockout muscle, two‐way ANOVA revealed significant alterations in the expression of *Bmal1*, *Per1*, *Per2*, and *Dbp*. Additionally, the amplitudes of these core clock genes were reduced (Figure 4B).

Analysis of differentially expressed genes (DEGs) at various time points revealed that ZT8 had the highest number of DEGs (907), while ZT2, ZT14, and ZT20 had 111, 95, and 284 DEGs, respectively (Figure 4C). To identify the target genes directly regulated by *Bmal1*, we examined both the DEGs and those whose rhythmic expression was lost following *Bmal1* knockout. This analysis revealed 270 rhythmic genes that showed differential expression (Figure 4D). Wikipathways enrichment analysis revealed convergence between pathways enriched for rhythm‐disrupted genes in aged mice and those enriched by the 270‐gene signature. Shared pathways comprised the MAPK signaling pathway, α6β4 integrin signaling pathway, EGFR1 signaling pathway, Focal adhesion, Focal adhesion‐PI3K‐AKT–mTOR signaling pathway, Insulin signaling pathway, and ROS‐associated pathways—specifically the Oxidative damage response pathway in aged mice and the Hypoxia‐dependent self‐renewal of myoblasts pathway in muscle‐specific *Bmal1* KO mice (Figure S6A,B). KEGG pathway enrichment analysis of these 270 genes also identified several enriched ROS‐related signaling pathways, including the TNF, HIF‐1, PI3K‐AKT, and MAPK signaling pathways (Figure 4E). In the HIF‐1 pathway, we observed that the rhythmic expression of the antioxidant *Hmox1* was significantly disrupted following *Bmal1* knockout, emerging as the most significantly altered target in our differential analysis (Figure 4F). Furthermore, although two‐way ANOVA revealed no significant changes in the overall 24‐h expression levels of other oxidative stress‐related genes, such as *Nrf2* and *Cat*, we observed a distinct reduction in their rhythmic amplitudes within the skeletal muscle of *Bmal1* KO mice (Figure 4G,H). Notably, the rhythmic expression of these antioxidant genes in the gastrocnemius muscle of control mice peaked at ZT14, the time when mice are most active at night. However, after *Bmal1* knockout, the expression of these genes at ZT14 was downregulated (Figure 4F–H). These findings suggest that *Bmal1* knockout in skeletal muscle cells reduces the number of rhythmic genes in the gastrocnemius muscle, diminishes the amplitude of core clock genes, and disrupts the rhythmic expression of antioxidant‐related genes such as *Hmox1*, *Nrf2*, and *Cat*. Consistent with this, the circadian RNA expression profiles of *Hmox1* and *Nrf2* in skeletal muscle from aged mice aligned with those in *Bmal1* KO mice relative to young controls (Figure S6C). Collectively, these findings indicate that *Bmal1* deficiency induces a rhythmic gene expression profile that partially resembles the aged phenotype.

To investigate how *Bmal1* regulates antioxidant gene expression, we knocked down *Bmal1* in C2C12 myotubes using ADV‐shRNA and measured the expression of the antioxidant genes *Hmox1* and *Nrf2*. Compared to the control group, *Bmal1* knockdown significantly reduced the expression of both *Hmox1* and *Nrf2* (Figure 4I–L). Next, to determine whether *Bmal1* directly regulates *Hmox1* transcription, we analyzed a 2 kb region of the *Hmox1* promoter and identified a BMAL1‐binding E‐box at positions 1957–1966 (Figure 4M). Additionally, a BMAL1‐binding signal was detected at this same region in the skeletal muscle ChIPseq database (Figure 4N). Through dual‐luciferase reporter assays, we found that cotransfection of *Bmal1* overexpression plasmid with the *Hmox1*‐WT plasmid significantly increased luciferase activity in 293 T cells. However, when we cotransfected the *Bmal1* overexpression plasmid with the *Hmox1*‐mutant plasmid (E‐box deletion), the transcriptional activation of luciferase by BMAL1 was abolished (Figure 4O). This suggests that BMAL1 binds to the E‐box element of the *Hmox1* promoter to regulate its transcription. Furthermore, analysis of ChIP‐seq databases revealed that both NRF2 and BMAL1 possess adjacent binding motifs and can cooccupy the *Hmox1* promoter region (Figure S7A). However, to determine if they form a collaborative regulatory complex, we performed co‐immunoprecipitation (Co‐IP) assays. The results showed no significant physical interaction between NRF2 and BMAL1 (Figure S7B), indicating that these two factors do not form a direct physical complex but rather independently regulate *Hmox1* transcription.

To further confirm whether *Bmal1* deletion in skeletal muscle cells inhibits *Hmox1* expression and reduces antioxidant capacity, we infected C2C12 myotubes with ADV‐shRNA for 4 days. After the infection, we measured intracellular ROS levels using DCFH‐DA. Compared to the control group, *Bmal1* knockdown significantly increased ROS levels in myotubes (Figure 4P–S).

Together, these results reveal that BMAL1 directly regulates the transcription of the antioxidant gene *Hmox1* and plays a key role in maintaining ROS homeostasis in skeletal muscle cells.

### 2.5 Skeletal Muscle *Bmal1* Regulates IL‐1α Expression via the Antioxidant Pathway

**FIGURE 5:**
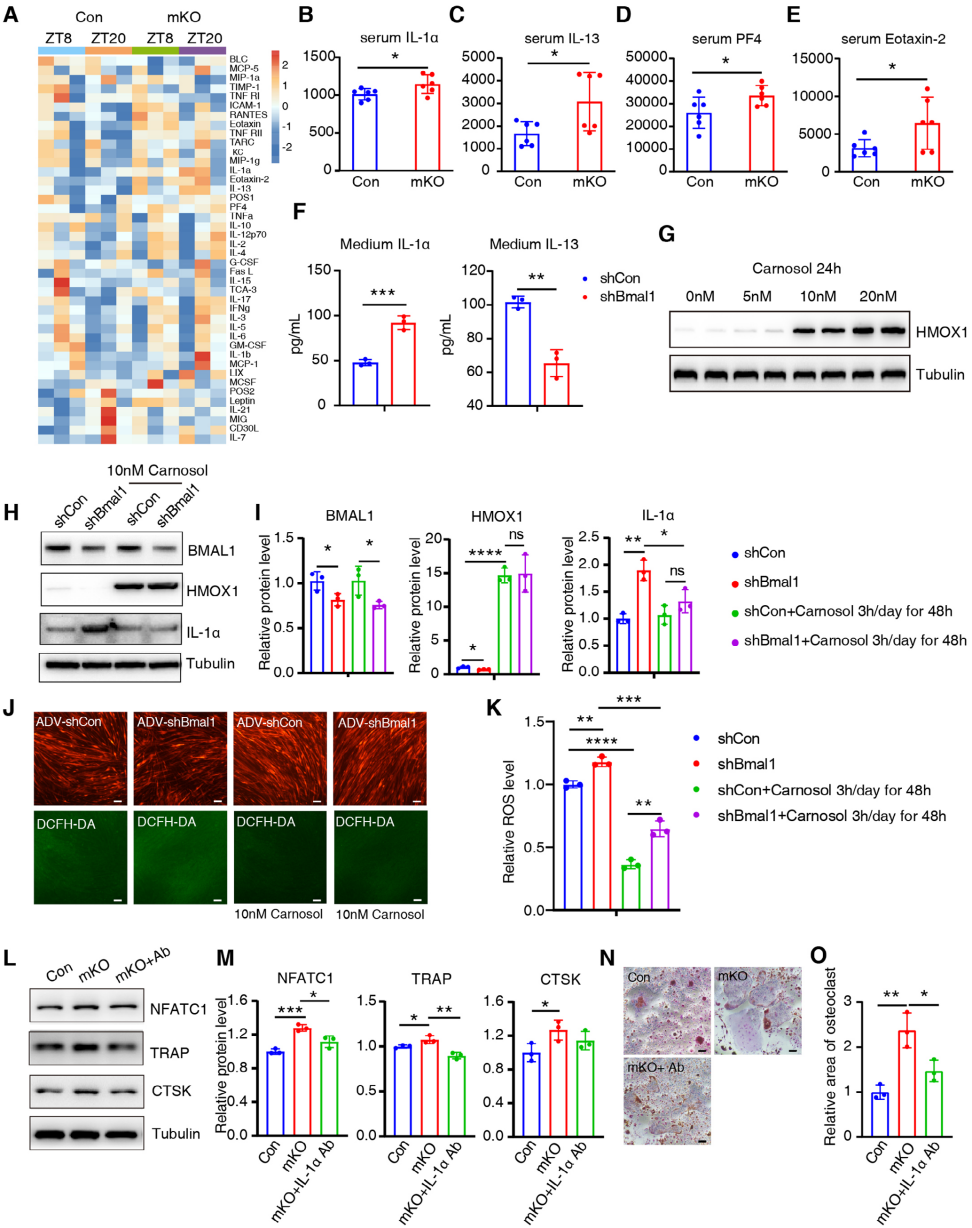
Skeletal muscle *Bmal1* deficiency promotes osteoclast differentiation via the HMOX1–IL‐1α axis. (A) Relative levels of 40 inflammatory cytokines in serum from mKO mice and littermate controls at ZT8 and ZT20, measured using a cytokine array. (B–E) Quantification of serum levels for selected cytokines: (B) IL‐1α, (C) IL‐13, (D) PF4 (CXCL4), and (E) Eotaxin‐2 (CCL24) in mKO and control mice. (F) Secreted levels of IL‐1α and IL‐13 in conditioned media from control and *Bmal1* knockdown (ADV‐shBmal1) C2C12 myotubes. (G) HMOX1 protein expression in control and *Bmal1* knockdown C2C12 myotubes treated with increasing concentrations of Carnosol (0, 5, 10, 20 μM). (H) Representative western blot of BMAL1, HMOX1, IL‐1α, and Tubulin in control and *Bmal1* knockdown C2C12 myotubes treated with 10 μM Carnosol (3 h/day for 2 consecutive days). (I) Quantification of protein levels from (H) for BMAL1, HMOX1, and IL‐1α (*n* = 3). (J) Representative fluorescence micrographs of intracellular reactive oxygen species (ROS) levels detected by DCFH‐DA staining in control and *Bmal1* knockdown C2C12 myotubes treated with 10 μM Carnosol (scale bar: 200 μm). (K) Quantification of intracellular ROS levels (*n* = 3). (L, M) Osteoclast differentiation markers: (L) Representative western blots and (M) quantification of NFATC1, TRAP and CTSK protein expression in cocultured BMMs under three conditions: (1) Coculture with CONTROL myotubes, (2) Coculture with *Bmal1* knockdown myotubes, (3) Coculture with *Bmal1* knockdown myotubes in the presence of a neutralizing anti‐IL‐1α antibody. (N) Representative TRAP staining of osteoclasts differentiated in the coculture conditions described for (L, M) (scale bar: 100 μm). (O) Quantification of TRAP‐positive multinucleated osteoclasts (MNCs) formed in the coculture conditions (*n* = 3). Data are presented as mean ± SD. *p < 0.05, **p < 0.01, ***p < 0.001, ****p < 0.0001, ns, not significant.

GO pathway enrichment analysis of DEGs showed that NF‐κB signal transduction was enriched (Figure S8), suggesting that changes in the expression of antioxidant genes may influence muscle inflammation‐related responses. To investigate how reduced antioxidant genes in skeletal muscle cells, resulting from *Bmal1* knockout, affect osteoclast differentiation, we conducted an inflammatory factor chip analysis. This analysis measured the expression of 40 inflammatory markers in the serum of control and muscle‐specific *Bmal1* knockout mice at ZT8 and ZT20 (Figure 5A). Among these inflammatory factors, several exhibited diurnal variations in control mice that were abolished in *Bmal1* knockout mice, including GM‐CSF, IL‐6, IL‐15, IL‐17, MCP‐1, IL‐21, and M‐CSF. Conversely, novel diurnal variation emerged in *Bmal1* KO mice for RANTES, TNF RI, and Leptin (Figure S9). Another subset showed no diurnal variation but exhibited elevated baseline expression levels in *Bmal1* KO mice (Figure S9), exemplified by IL‐1α, IL‐13, PF4, and Eotaxin‐2. Analysis of overall expression levels confirmed that serum concentrations of IL‐1α, IL‐13, PF4, and Eotaxin‐2 were significantly elevated in *Bmal1* KO mice compared to controls (Figure 5B–E). While previous studies indicate that both IL‐1α and IL‐13 can be secreted by skeletal muscle cells under certain physiological conditions (Authier et al. 1999; Scheler et al. 2013), our in vitro experiments revealed a divergent response to circadian disruption. When we knocked down *Bmal1* in C2C12 myotubes, we observed a significant increase in IL‐1α levels in the cell supernatant, whereas IL‐13 levels decreased significantly (Figure 5F). These findings suggest that the elevated serum IL‐13 observed in vivo does not originate directly from the *Bmal1*‐deficient skeletal muscle. Instead, IL‐1α is one of the pro‐inflammatory factors directly released by the muscle, playing a key role in regulating osteoclast differentiation.

Carnosol, an activator of HMOX1, interacts with HMOX1 through hydrogen bonding and hydrophobic forces (Chu et al. 2024). To determine whether the increase in IL‐1α expression associated with *Bmal1* knockout is linked to *Hmox1* expression, we treated C2C12 myotubes with various concentrations of Carnosol. The results showed that 5 nM, 10 nM, and 20 nM Carnosol activated HMOX1 expression in a dose‐dependent manner (Figure 5G). When we treated myotubes with 10 nM Carnosol for 3 h daily over two consecutive days, we observed that Carnosol significantly inhibited the increase in IL‐1α expression induced by *Bmal1* knockdown (Figure 5H,I). Simultaneously, we measured intracellular ROS levels in myotubes and found that *Bmal1* knockdown markedly elevated ROS, whereas Carnosol treatment significantly reduced ROS levels in both wild‐type and *Bmal1* knockdown myotubes (Figure 5J,K). These results suggest that HMOX1 can mitigate the inflammatory response triggered by *Bmal1* deletion, highlighting the role of antioxidant signaling in maintaining inflammation homeostasis in muscle cells.

Additionally, we performed primary cell coculture experiments and found that IL‐1α antibody treatment could reverse the effects of muscle‐specific *Bmal1* knockout on osteoclast differentiation. Specifically, IL‐1α antibody treatment reduced the expression of osteoclast differentiation markers, including NFATC1, TRAP, and CTSK, that were upregulated due to *Bmal1* knockout (Figure 5L,M). TRAP staining and subsequent quantitative analysis further confirmed that the IL‐1α neutralizing antibody significantly inhibited the osteoclast hyperactivation caused by myocyte‐specific *Bmal1* deletion. Specifically, coculture with *Bmal1*‐deficient myocytes increased osteoclast differentiation by an average of 2.37‐fold compared to the control group. However, following IL‐1α antibody intervention, this pathological enhancement was substantially reversed, with osteoclast differentiation returning toward baseline levels (1.47‐fold of the control) (Figure 5N,O). These quantitative results indicate that muscle‐derived IL‐1α is an important inflammatory mediator contributing to osteoclastogenesis in this model.

Previous studies have shown that *Bmal1* knockdown promotes the secretion of IL‐1β in macrophages (Early et al. 2018), suggesting it may also act as a potential regulator of bone metabolism. Therefore, we validated the regulation of IL‐1α and IL‐1β by BMAL1 through both in vitro and in vivo experiments. At the RNA level, *Bmal1* knockdown in C2C12 myotubes decreased the expression of both *Il1a* and *Il1b* (Figure S10A); however, at the protein level, both IL‐1α and IL‐1β were upregulated (Figure S10C,D). Furthermore, similar to IL‐1α, the level of secreted IL‐1β in the myotube culture medium was also increased (Figure S10B). In vivo, we assessed the protein levels of IL‐1α and IL‐1β in muscle tissues at ZT8 and ZT20. The results showed that in the knockout group, the levels of IL‐1α were significantly elevated at both ZT8 and ZT20, whereas IL‐1β showed no obvious alterations (Figure S10E,F). These results suggest that *Bmal1* knockdown may promote the translation and secretion of the IL‐1 family in vitro. However, in the in vivo environment, *Bmal1* deficiency enhances the expression of IL‐1α, suggesting that in vivo, muscle‐derived IL‐1α accumulates more prominently than IL‐1β. While our in vitro data show that *Bmal1* deficiency can enhance the translation and secretion of both IL‐1 family members despite decreased mRNA levels, research has indicated that IL‐1β does not exert a direct regulatory effect on the osteoclast differentiation of BMMs (Levescot et al. 2021). Thus, the stimulatory effect observed in our coculture system is likely driven more strongly by IL‐1α. Collectively, these results indicate that muscle‐specific *Bmal1* knockout induces systemic dysregulation of serum inflammatory cytokines. Skeletal muscle contributes to this inflammatory milieu by releasing IL‐1α following the modulation of *Hmox1* expression, acting as a key—though likely not exclusive—mediator of enhanced osteoclast differentiation.

### 2.6 Time‐Restricted Feeding Increases Bone Mass in Skeletal Muscle‐Specific *Bmal1* Knockout and Aged Mice

**FIGURE 6:**
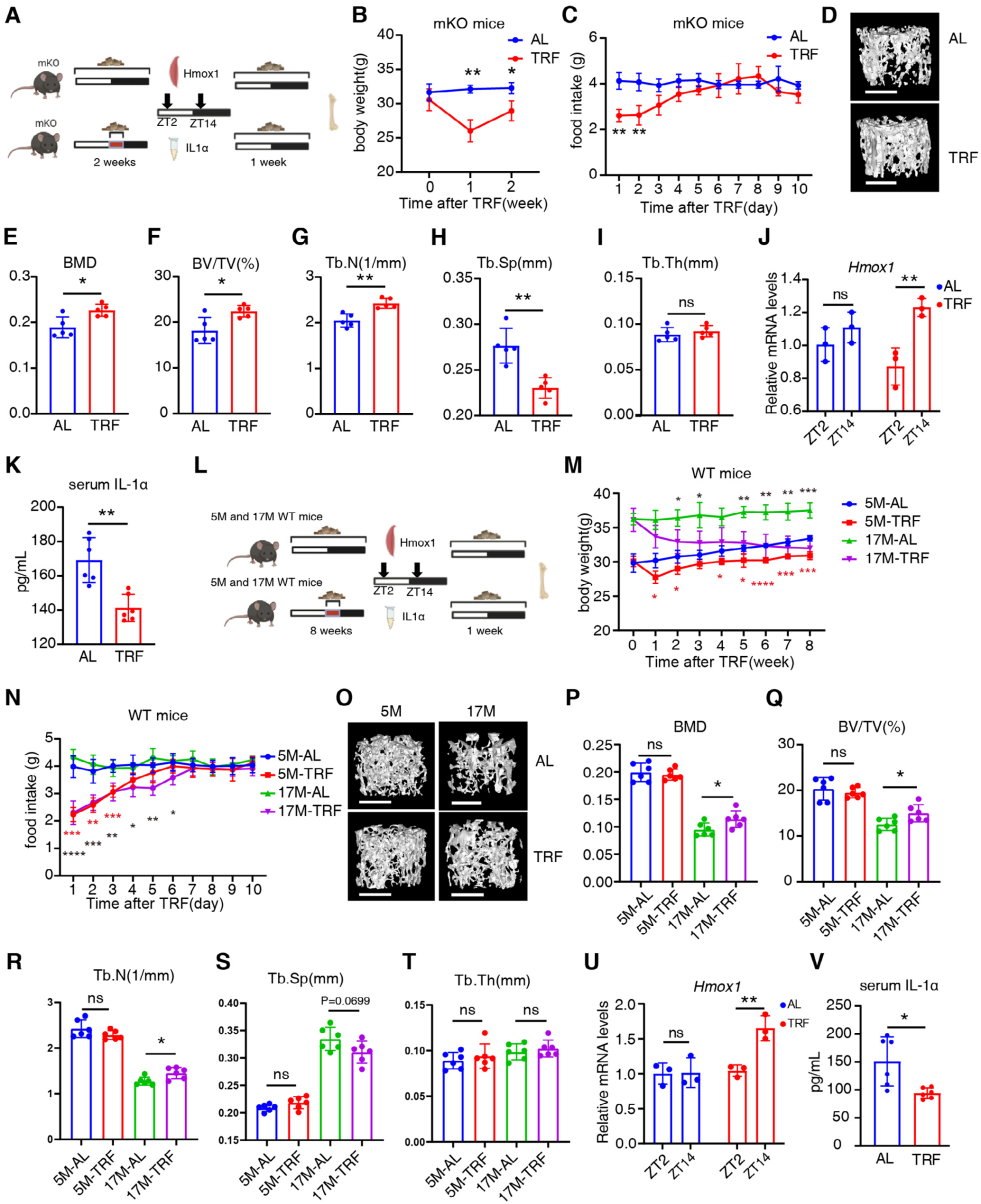
Time‐restricted feeding (TRF) rescues bone loss in skeletal muscle‐specific *Bmal1* knockout and aged male mice. (A) Schematic of the TRF protocol for mKO mice. Gastrocnemius muscle and serum were collected at ZT2 and ZT14 post‐TRF. Muscle *Hmox1* mRNA was analyzed at each time point; serum samples from ZT2/ZT14 were pooled for IL‐1α measurement. Femora were harvested 1 week post‐TRF. (B, C) Body weight and food intake changes in *ad libitum* (AL) and ZT12‐16 TRF mKO mice (*n* = 5). (D) Representative 3D micro‐CT reconstructions of distal femora from AL and TRF mKO mice (scale bar: 1 mm). (E–I) Quantitative micro‐CT analysis of trabecular bone parameters: (E) BMD, (F) BV/TV, (G) Tb.N, (H) Tb.Sp, (I) Tb.Th in mKO mice (*n* = 5). (J) Relative *Hmox1* mRNA expression in gastrocnemius muscle at ZT2 and ZT14 in AL and TRF mKO mice (*n* = 3 per time point). (K) Serum IL‐1α levels (pooled ZT2/ZT14 samples) in AL and TRF mKO mice (*n* = 6). (L) Schematic of the TRF protocol for 5 and 17‐month‐old wild‐type mice. (M, N) Body weight and food intake changes in AL and TRF 5 and 17‐month‐old mice (*n* = 6, red asterisks indicate comparisons between the TRF and AL groups in 5‐month‐old mice, while black asterisks indicate comparisons between the TRF and AL groups in 17‐month‐old mice). (O) Representative 3D micro‐CT reconstructions of distal femora from young and aged AL and TRF‐fed mice (scale bar: 1 mm). (P–T) Quantitative micro‐CT analysis of young and aged trabecular bone: (P) BMD, (Q) BV/TV, (R) Tb.N, (S) Tb.Sp, (T) Tb.Th (*n* = 6). (U) Relative *Hmox1* mRNA expression in gastrocnemius muscle at ZT2 and ZT14 in aged (17‐month‐old) mice (*n* = 3 per time point). (V) Serum IL‐1α levels (pooled ZT2/ZT14 samples) in aged mice (*n* = 6). Data presented as mean ± SD. *p < 0.05, **p < 0.01, ***p < 0.001, ****p < 0.0001, ns, not significant.

TRF can effectively entrain peripheral clocks and mitigate metabolic desynchrony (Chaix et al. 2014; Manoogian and Panda 2017; Zarrinpar et al. 2016), even in skeletal muscle lacking central clock control (Kumar et al. 2024). We hypothesized that driving systemic diurnal metabolic rhythms through TRF could rescue bone loss in muscle‐specific *Bmal1* knockout mice. Following a 1‐week acclimation, mice were assigned to either *ad libitum* (AL) feeding or a specific ZT12‐16 TRF schedule. This particular window was selected because initiating TRF at ZT12 optimally aligns with the murine active phase (Regmi et al. 2021). Furthermore, to successfully entrain the clock‐deficient muscle, we utilized a compressed 4‐h feeding duration to maximize the fasting–feeding amplitude, serving as a potent systemic zeitgeber previously validated by our group (Zhai et al. 2022). After 2 weeks, the mice were returned to a free‐feeding regimen for another week, and femoral tissues were collected (Figure 6A). We found that TRF at ZT12‐16 significantly reduced body weight compared to mice fed AL (Figure 6B), and during the initial 2 days of the dietary intervention, food intake in the TRF group was significantly lower than that of the AL control group (Figure 6C). Micro‐CT analysis revealed that TRF at ZT12‐16 increased cancellous bone density, BV/TV, and the number of trabeculae while reducing trabecular space (Figure 6D–6I). Further investigation of *Hmox1* expression in the gastrocnemius muscle by qPCR revealed that TRF at ZT12‐16 drove a feeding‐induced diurnal variation in *Hmox1* expression in muscle‐specific *Bmal1* knockout mice (Figure 6J). Additionally, serum IL‐1α levels significantly decreased after TRF treatment (Figure 6K).

These results indicate that TRF at ZT12‐16 can improve bone mass loss and trabecular bone microstructural damage induced by muscle *Bmal1* knockout. It also enhances the rhythmic expression of the antioxidant gene *Hmox1* in skeletal muscle and reduces systemic IL‐1α levels.

We also acclimated 5 and 17‐month‐old mice to a light/dark cycle for 1 week before subjecting them to ZT12‐16 TRF or AL. After 8 weeks, the mice were returned to a free‐feeding regimen for one more week, and femoral tissues were collected (Figure 6L). We observed that TRF significantly reduced body weight compared to AL in both 5‐ and 17‐month‐old mice (Figure 6M); furthermore, food intake in the TRF groups was significantly lower than that of the control groups during the first 3 and 6 days of the dietary intervention, respectively (Figure 6N). Micro‐CT analysis showed that TRF increased cancellous BMD, BV/TV, and the number of trabeculae (Figure 6O–6R), while trabecular spacing and thickness remained unchanged (Figure 6S,T) in 17‐month‐old mice. However, in 5‐month‐old mice, 8 weeks of TRF had no significant effect on bone mineral density or trabecular bone parameters (Figure 6O–6T). TRF also drove a feeding‐induced diurnal *Hmox1* expression in aged muscle (Figure 6U). Serum IL‐1α levels significantly decreased following TRF treatment compared to AL aged mice (Figure 6V). Furthermore, we collected muscle tissue samples at ZT2 and ZT14 on the final day of the TRF intervention. RNA‐seq analysis revealed that at ZT14, TRF induced the upregulation of 169 genes and the downregulation of 95 genes in the muscle, and TRF resulted in 686 upregulated and 225 downregulated genes at ZT2 (Figure S11A). Subsequent pathway enrichment analysis of these DEGs highlighted the enrichment of classical cell proliferation‐ and aging‐related signaling pathways, including the p53, AMPK, FoxO, and PI3K‐Akt pathways. Concurrently, the circadian entrainment pathway was also significantly enriched, suggesting that TRF may modulate muscle biological rhythms and subsequently influence muscle aging (Figure S11B).

These results indicate that TRF at ZT12‐16 enhances the rhythmic expression of the antioxidant gene *Hmox1* in aged skeletal muscle, reduces systemic IL‐1α levels, and ultimately alleviates bone loss in aged mice.

## 3 Discussion

The age‐related decline of the musculoskeletal system is a multifaceted process involving the parallel deterioration of both muscle and bone. The frequent coexistence of sarcopenia and osteoporosis, termed osteosarcopenia, has led to increased recognition that these tissues may influence each other's aging trajectories (Gielen et al. 2023). Indeed, individuals with osteosarcopenia experience higher fracture rates than those with osteoporosis alone (Teng et al. 2021), suggesting that aging muscles may actively contribute to the regulation of bone health. However, the molecular mediators of this muscle‐bone crosstalk, and particularly the role of the skeletal muscle circadian clock, have remained unclear. In this study, we reveal that aging is accompanied by disrupted circadian coupling between muscle and bone, and that specific deletion of the core clock gene *Bmal1* in skeletal muscle recapitulates aging‐related osteoporosis in mice.

Regarding our age‐specific mouse models, while overall skeletal maturity in male C57BL/6 mice peaks at 5–6 months, trabecular bone volume—our primary phenotypic readout—peaks earlier at 1–2 months before gradually declining (Glatt et al. 2007). Thus, 2‐month‐old mice provided an accurate presenescent, peak‐trabecular baseline for our transcriptomic screening. To exclude growth‐related confounders, we explicitly incorporated mature and older adult cohorts into our downstream phenotypic and functional assays. Notably, mKO mice developed severe trabecular osteopenia by 4 months of age, which progressively deteriorated by 10 months of age (Figure S5), indicating premature pathological bone loss. Furthermore, TRF rescued bone loss in 17‐month‐old mice but not in 5‐month‐old mature mice, confirming that this muscle‐bone mechanism specifically ameliorates age‐related catabolism rather than altering physiological peak bone homeostasis.

Our temporal RNA‐seq revealed an unexpected acquisition of 1711 newly rhythmic genes in aged bone. While aging typically dampens global circadian rhythms (Wolff et al. 2023), it is notable that the number of rhythmic genes can also increase in different tissues under aging or environmental stimuli (Chen et al. 2016; Sato et al. 2017). As the intrinsic core clock dampens, the resulting loss of local transcriptional repression likely increases the tissue's reliance on systemic diurnal cues (Koronowski et al. 2019). Consequently, altered inflammatory cytokines and remodeled muscle‐bone crosstalk may act as systemic drivers to synchroni these de novo rhythmic genes to adapt to the aged microenvironment.

A previous study has shown that in BMMs, BMAL1 directly binds to the promoter of the key antioxidant gene *Nrf2*, regulating its rhythmic expression and suppressing IL‐1β production (Early et al. 2018). NRF2 senses intracellular redox status and controls downstream antioxidant gene expression via antioxidant response elements (ARE), thereby generating rhythmic redox signaling (Wible et al. 2018). In mouse liver (Kondratov et al. 2006), neurons (Musiek et al. 2013), and fibroblasts (O'Neill and Feeney 2014), *Bmal1* knockout increases ROS, underscoring its role in redox homeostasis. Consistent with these findings, our study showed that *Bmal1* knockdown in C2C12 myotubes elevated intracellular ROS, further confirming BMAL1 as a key regulator of cellular redox balance.

Additionally, we found that BMAL1 more significantly regulates the rhythmic expression of *Hmox1* compared to *Nrf2*, likely due to *Hmox1* being controlled by both BMAL1 and NRF2. Beyond direct transcriptional regulation, the circadian clock integrates with mitochondrial function to maintain ROS homeostasis by synchronizing mitochondrial ROS production with antioxidant defense mechanisms (Mezhnina et al. 2022). Moreover, in both humans and green algae, peroxiredoxin enzyme activity follows rhythmic cycles independent of transcription, suggesting that metabolite‐driven periodic ROS regulation works alongside the TTFL of the molecular circadian clock (Bass and Takahashi 2011). Thus, the biological clock and redox homeostasis are mutually regulated. Elevated ROS levels may activate inflammatory responses via the NF‐κB signaling pathway, promoting inflammatory cytokine release (Bhattacharyya et al. 2014). In our model, we observed a robust phenotypic link between *Bmal1* deficiency, severe ROS accumulation, transcriptomic enrichment of NF‐κB signaling, and IL‐1α secretion. However, we did not provide direct experimental evidence of NF‐κB activation (such as p65 phosphorylation or nuclear translocation), nor did we perform functional validation using NF‐κB inhibitors. Therefore, we must explicitly state that the proposed ROS–NF‐κB–IL‐1α axis remains a hypothetical and inferential mechanism in our study, rather than a directly demonstrated conclusion. Further detailed molecular studies are required to definitively establish whether IL‐1α induction is strictly dependent on NF‐κB activation in this specific skeletal muscle context. Restoring *Bmal1* expression in muscle tissue led to a systemic reduction in inflammatory markers across peripheral tissues, including the liver, lung, and white adipose tissue (Miguel A. Gutierrez‐Monreal et al. 2024), highlighting *Bmal1* as a key regulator of inflammatory pathways.

The feeding/fasting cycle's periodicity entrains antioxidant defense rhythms, akin to other circadian metabolic processes (Mezhnina et al. 2022). TRF has been shown to reduce inflammation, oxidative stress, and immune cell infiltration in both diet‐induced obesity and hepatic ischemia–reperfusion injury models in mice (Zeb et al. 2021). Our study also found that night‐time TRF reshaped the rhythmic expression of *Hmox1* in mouse muscle and decreased serum IL‐1α levels, further supporting TRF as a nonpharmacological approach to reduce inflammation and mitigate bone loss associated with inflammation. Clinical studies have reported that 12 weeks of TRF can increase BMC in obese individuals and slow the decline in the bone formation marker P1NP (Lobene et al. 2021). In contrast, 6 weeks of TRF in older adults had no impact on body mass, lean mass, or BMD (Martens et al. 2020). In our previous study, a 2‐week ZT12‐16 TRF intervention in 2‐month‐old male mice showed no significant changes in bone mineral density or trabecular microstructure parameters (Ye et al. 2024). This may be attributed to the robust bone metabolism in young mice, which could render them less responsive to dietary interventions. Therefore, the effects of TRF on bone metabolism may depend on factors such as the duration of the intervention and the characteristics of the target population, including age and obesity status, and long‐term TRF interventions may have clinical potential for inhibiting age‐related bone loss.

In mice with muscle‐specific *Bmal1* knockout, serum levels of various inflammatory factors, including IL‐1α, IL‐13, PF4, and Eotaxin‐2, were significantly elevated compared to control mice. IL‐1α promotes osteoclast differentiation by enhancing the expression of MC‐SF and PGE in osteoblasts, while reducing their OPG expression (Tanabe et al. 2005). Numerous studies have linked the activity of inflammatory factors in the IL‐1 family to bone diseases, and targeting these cytokines has shown potential for improving bone loss‐related conditions (Tseng et al. 2022). IL‐13 is known to play a critical role in the anti‐inflammatory response (Kolosowska et al. 2019; Van Dyken and Locksley 2013). After knocking down *Bmal1* in C2C12 myotubes, we observed a definitive decrease in local IL‐13 secretion. This in vitro reduction, contrasted with the in vivo serum elevation, indicates that the circulating IL‐13 in muscle *Bmal1* knockout mice does not originate from the skeletal muscle itself. Rather, it likely represents a secondary, systemic compensatory response from peripheral immune cells (such as Th2 cells or alternatively activated macrophages). PF4 has been implicated in bone loss by inhibiting the integrin α5‐FAK‐ERK pathway (Li et al. 2023). In human monocytes, PF4 synergizes with TLR8 to activate TBK1‐IRF5 and induce inflammation (Yang et al. 2022), suggesting that PF4 may directly regulate osteoclast differentiation by activating inflammatory pathways. The expression of eotaxin‐1 and eotaxin‐2 is elevated in individuals with osteopenia and osteoporosis compared to healthy controls (Ahmadi et al. 2020). Eotaxin‐1 promotes osteoclast migration and differentiation (Kindstedt et al. 2017). However, it remains unclear whether eotaxin‐2 directly influences osteoclast differentiation.

It may seem paradoxical that a prooxidant and IL‐1α‐enriched environment induces bone loss without causing muscle atrophy. However, this phenotypic divergence is explained by concurrent *Bmal1*‐mediated alterations in myogenic signaling that override the catabolic effects of inflammation. Specifically, our RNA‐seq data revealed a downregulation of myostatin (*Mstn*) in *Bmal1* KO muscle. Myostatin is a potent muscle growth inhibitor under circadian control, and its downregulation drives compensatory muscle preservation and lean mass increase (Liu et al. 2025). Consequently, while the muscle is protected by this myostatin‐deficiency‐driven hypertrophy, the secreted inflammatory factors remain highly detrimental to the sensitive adjacent trabecular bone.

A methodological consideration is our use of the tibia for temporal RNA‐seq and the distal femur for micro‐CT. This strategy optimizes technical readouts: the distal femur is the gold standard for assessing trabecular microarchitecture, whereas the tibia yields the high‐quality RNA essential for circadian transcriptomics. Importantly, both are hindlimb long bones enveloped by skeletal muscle, sharing a highly similar biochemical microenvironment. Our findings indicate that muscle *Bmal1* deficiency upregulates the translation and secretion of IL‐1α. While skeletal muscle may not be the sole source of elevated circulating cytokines, muscle‐derived IL‐1α acts as a significant contributor to the pro‐osteoclastogenic environment via both systemic circulation and local paracrine diffusion at the muscle‐bone interface. Because systemic factors alone cannot fully explain the site‐specific nature of age‐related bone loss—such as distinct osteoclastogenic patterns between femoral and vertebral sites (Almeida et al. 2007; Farr et al. 2017)—this highly concentrated local paracrine gradient is critical. Consequently, both sites are exposed to the same IL‐1α‐enriched catabolic drivers, which preferentially affect the rapidly turning‐over cancellous bone over cortical bone, ensuring the tibial transcriptome closely parallels the mechanisms underlying femoral bone loss. A limitation of our TRF intervention is that mice in the restricted feeding groups exhibited significantly reduced initial food intake and overall lower body weight compared to the *ad libitum* controls. This discrepancy introduces critical confounding factors. First, given the abundant evidence that body weight‐induced mechanical loading positively regulates bone mass (Kitase et al. 2022), the reduced body weight in the TRF group complicates the interpretation of its net therapeutic effect on age‐related bone loss. Furthermore, we cannot completely rule out the possibility that mild caloric restriction‐induced mechanisms, such as the activation of bone tissue autophagy (Jamshed et al. 2019; Ulgherait et al. 2021; Zhu et al. 2022), also synergistically contributed to the observed bone mass improvements. Future studies utilizing strict pair‐fed isocaloric controls will be necessary to definitively uncouple the benefits of circadian entrainment from those of caloric deficit.

## 4 Conclusions

This study identified remodeling of the rhythmic interaction between skeletal muscle and bone tissue in the context of aging in mice. We constructed muscle‐specific *Bmal1* knockout mice to investigate the mechanism by which age‐related decline of muscle *Bmal1* leads to bone loss. We found that *Bmal1* deficiency can directly suppress the rhythmic expression of the antioxidant gene *Hmox1*, thereby increasing intracellular ROS levels in muscle cells and inducing the expression of the inflammatory cytokine IL‐1α, which ultimately promotes osteoclast differentiation and leads to bone loss. Through TRF interventions in both *Bmal1* knockout and aged mice, we found that TRF drove a feeding‐induced diurnal expression of muscle *Hmox1*, reduced serum IL‐1α levels, and ultimately alleviated bone mass. These findings indicate that TRF could serve as an effective nonpharmacological intervention strategy to mitigate age‐related bone loss.

## 5 Materials and Methods

### 5.1 Animals

CKMM‐cre and *Bmal1* flox mice were obtained from Dr. Ying Xu's lab at the Cambridge‐Soochow Genomic Resource Center, Soochow University. Skeletal muscle‐specific *Bmal1* knockout mice were generated by crossing *Bmal1* flox^+/+^ mice with CKMM‐cre^+/−^ *Bmal1* flox^+/+^ mice. CKMM‐cre^−/−^ *Bmal1* flox^+/+^ mice were used as controls. All mice were housed in an SPF animal facility under a 12‐h LD cycle. The wheel‐running activity of the mice was recorded and analyzed using the ClockLab system (Actimetrics, Evanston, IL). In the time‐restricted feeding (TRF) experiment, mice were randomly divided into experimental and control groups. All animal procedures were approved by the Animal Care and Use Committee of the CAM‐SU Genomic Resource Center, Soochow University (YX‐2021–2).

### 5.2 Body Composition Analysis

4‐month‐old muscle‐specific *Bmal1* knockout and control mice were weighed and subjected to body composition analysis using an LF50 body composition analyzer. The measuring chamber was cleaned before each measurement to ensure a dry environment. Body composition assessment comprised three key parameters: fat mass, lean mass, and free body mass.

### 5.3 Immunohistochemistry

Fresh gastrocnemius muscle tissue from mice was collected and fixed for 24 h. The tissue was then embedded in paraffin and sectioned. The paraffin sections were deparaffinized to water, followed by antigen retrieval using Tris‐EDTA buffer. To block endogenous peroxidase activity, 3% hydrogen peroxide was added and incubated at room temperature in the dark for 25 min, followed by PBS washing. The sections were then blocked with 3% BSA at room temperature for at least 30 min. Primary antibodies, including anti‐P21 (Affinity, 1:500), were added and incubated overnight at 4°C. After PBS washing, HRP‐labeled secondary antibodies corresponding to the primary antibodies were applied and incubated at room temperature for 50 min. The sections were washed three times with PBS and developed using DAB. Hematoxylin was used for nuclear counterstaining. Finally, the sections were mounted with neutral resin, and images were captured.

### 5.4 Protein Extraction and Western Blotting

Cells or *gastrocnemius muscles were* lysed in RIPA buffer containing protease inhibitors and incubated on ice for 30 min. The subsequent steps were identical to the tissue protein extraction procedure. Protein samples were mixed with 5× loading buffer and denatured at 100°C for 5 min. After SDS‐PAGE electrophoresis, proteins were transferred onto PVDF membranes and blocked with 5% milk. The membranes were incubated overnight at 4°C with primary antibodies (anti‐BMAL1: Proteintech 14,268–1‐AP 1:1000, anti‐NFATC1: Affinity DF6446 1:1000, anti‐TRAP: Affinity DF6989 1:1000, anti‐CTSK: Daige db8038 1:1000, anti‐HMOX1: Daige db3474 1:1000, anti‐IL‐1α: Epizyme R015789 1:1000, anti‐IL‐1β: Epizyme R015789 1:1000, anti‐GAPDH: Proteintech 60,004–1‐Ig 1:3000, anti‐Tubulin: Proteintech 11,224–1‐AP 1:3000) at appropriate dilutions. After washes with TBST three times, membranes were incubated with secondary antibodies (Proteintech SA00001‐1, 1:10000; Proteintech SA00001‐2, 1:10000) at room temperature for 1 h. Bands were visualized using a chemiluminescence imager (Tannon), and protein levels were quantified using ImageJ software.

### 5.5 Micro‐CT Analysis

Unilateral femurs were fixed in 4% PFA and transferred to 70% ethanol for storage. Samples were scanned using a Bruker SkyScan 1176 micro‐CT scanner at 50 kV, 500 μA, 1000 ms, with a voxel size of 9 μm. For trabecular measurements, a 1.75‐mm region of interest, starting 540 μm proximal to the distal growth plate, was analyzed. Bone microstructural parameters, including bone mineral density (BMD), bone volume fraction (BV/TV), trabecular thickness (Tb.Th), trabecular number (Tb.N), and trabecular separation (Tb.Sp), were calculated. Materialize Magics 24.0 software was used to generate 3D visualizations of cortical and trabecular bone structures.

### 5.6 Von Kossa Staining

Mouse femoral hard tissue sections were fixed in 100% acetone for 10 min, repeated twice. The sections were then infiltrated with 70% ethanol for 3 min and placed in Wahaha purified water for at least 3 min. For staining, the sections were sequentially immersed in VK1 solution for 5 min and washed with double‐distilled water (ddH_2_O) for 2 min, followed by immersion in VK2 solution for 5 min with a gentle ddH_2_O wash for 2 min. The process was repeated with VK3 solution under the same conditions. For counterstaining, the sections were treated with Gleson solution containing acidic fuchsine for 30 min, briefly rinsed in 1% acetic acid for 5 s, and washed in ddH_2_O for 5 s. After air‐drying, the sections were dehydrated through sequential immersion in 90% ethanol and 100% ethanol. The dehydration process was completed with one treatment of ethanol/toluene solution and two treatments of pure toluene. Finally, the sections were mounted with a coverslip.

### 5.7 Toluidine Blue Staining

Mouse femoral sections were fixed in 100% acetone for 10 min, repeated twice. The sections were then infiltrated with 70% ethanol for 3 min and placed in Wahaha purified water for at least 3 min. Drop toluidine blue staining solution on the sections and stain for 20–30 min. Wash gently with running water to remove excess stain. Perform color differentiation using 95% ethanol under a microscope to control the differentiation effect. Soak in anhydrous ethanol for 1 min, followed by three 1–2 min dehydration steps using xylene, and finally mount with neutral balsam. Cartilage and osteoblasts appear blue‐purple, while the background is light blue.

### 5.8 Bone Tissue TRAP Staining

Mouse femoral sections were fixed in 100% acetone for 10 min, repeated twice. The sections were then infiltrated with 70% ethanol for 3 min and placed in Wahaha purified water for at least 3 min. Place the sections in 0.2 M Tris–HCl buffer and incubate at 37°C for 1 h. Subsequently, place the sections in TRAP staining solution and monitor under a microscope for TRAP‐positive changes. Stop the reaction at the appropriate time and wash with ddH_2_O. After air drying, dehydrate by using xylene for transparency and mount with a cover slip.

### 5.9 BMMs Culture Coculture and Osteoclast Differentiation

Primary BMMs were isolated from the femurs and tibias of 2‐month‐old C57BL/6 mice. Briefly, after excising both ends of the bones, the bones were placed into 200 μL pipette tips nested within 1.5 mL Eppendorf tubes and briefly centrifuged to collect the bone marrow. The marrow was resuspended in serum‐free α‐MEM and filtered through a 70‐μm cell strainer. Following centrifugation, the cell pellet was resuspended in α‐MEM supplemented with 10% FBS and incubated. After 20 h, the supernatant containing nonadherent cells was collected and further cultured in a medium supplemented with 30 ng/mL M‐CSF (Novoprotein, CB34). Once the BMMs reached confluence, they were washed with cold PBS, gently detached using a cell scraper, and subcultured into culture plates. On the following day, upon reaching approximately 30% confluence, osteoclastogenesis was induced by supplementing the medium with 30 ng/mL M‐CSF and 100 ng/mL RANKL (Novoprotein, C28A). Change the medium every other day. On day six, osteoclast differentiation can be clearly observed. Fix the cells with 2.5% glutaraldehyde for 10 min, wash thoroughly with distilled water, incubate in TRAP staining solution (Beyotime, P0332) at 37°C for 30–60 min, wash again with distilled water, and take pictures under a microscope. For coculture experiments, primary myocytes were seeded in the upper chamber of a transwell system, while BMMs were plated in the lower chamber.

### 5.10 BMSC Culture Coculture and Osteogenic Differentiation

Mouse BMSCs were purchased from Procell Life Science & Technology Co. Ltd. (Wuhan, China). Cells were cultured in α‐MEM supplemented with 10% FBS and 1% penicillin/streptomycin. For coculture experiments, primary myocytes were seeded in the upper chamber of a transwell system, while BMSCs were plated in the lower chamber. Upon reaching 90% confluence, BMSCs were induced with osteogenic differentiation medium (α‐MEM containing 10% FBS, 10 mM β‐Glycerophosphate disodium salt hydrate (Abmole, M3837), 50ug/ml VC (Abmole, M3121), and 10 nM dexamethasone (MCE, HY‐14648)). The medium was replaced every 2 days. For the mineralization assay, after 14 days of induction, the cells were fixed with 4% paraformaldehyde and stained with Alizarin Red S solution (OriCell, ALIR‐10001) to evaluate extracellular matrix calcium deposits.

### 5.11 RNA‐Seq and Rhythm Gene Analysis

The gastrocnemius muscle and tibia were collected and quick‐frozen with liquid nitrogen and stored at −80°C. Grind the muscle and bone tissue into a powder using liquid nitrogen and collect into an EP tube. Then, add 1 mL of Trizol for RNA extraction. RNA‐seq was performed on an Illumina NovaSeq 6000 platform at Shenggong, Shanghai, China. All samples (*n* = 3 per time point per age group) were sequenced in a single batch to eliminate technical batch effects. Raw read counts were normalized using DESeq2 to account for library size and composition. Prior to rhythmicity analysis by Metacycle, we filtered out genes with an FPKM of zero at all time points. The peak expression time (acrophase) for each rhythmic gene was mathematically calculated as a continuous variable (0–24 h) by the MetaCycle algorithm. For the phase distribution analysis and visualization (polar histograms), these calculated phase values were grouped into 2‐h bins. To ensure the identified rhythmic genes were not artifacts of low‐expression noise, a sensitivity analysis was performed by evaluating genes with an average FPKM < 1, which confirmed that the age‐related change in rhythmic genes was driven by robustly expressed transcripts. DESeq2 was used to identify DEGs. A *p* < 0.05 was considered significant. KEGG and Wikipathway enrichment analysis was performed using the Ouyi Cloud platform.

### 5.12 q‐PCR

RNA concentration was determined by NanoDrop (Thermo), and cDNA was synthesized using Plus All‐in‐one 1st Strand cDNA Synthesis SuperMix (Novoprotein, E047). Quantitative real‐time PCR was performed using NovoStart SYBR qPCR SuperMix Plus (Novoprotein, E096) with LightCycler 480II (Roche). The relative levels of *Trap*, *Ctsk*, *Bmal1*, *Hmox1*, and *Nrf2* were normalized to *Gapdh*. The q‐PCR primer sequences are as follows.

*Trap*‐forward: GCGACCATTGTTAGCCACATACG, *Trap*‐reverse: CGTTGATGTCGCACAGAGGGAT, *Ctsk*‐forward: AGCAGAACGGAGGCATTGACTC, *Ctsk*‐reverse: CCCTCTGCATTTAGCTGCCTTTG, *Alp*‐forward: CCAGAAAGACACCTTGACTGTGG, *Alp*‐reverse: TCTTGTCCGTGTCGCTCACCAT, *Runx2*‐forward: CCTGAACTCTGCACCAAGTCCT, *Runx2*‐reverse: TCATCTGGCTCAGATAGGAGGG, *Bglap*‐forward: GCAATAAGGTAGTGAACAGACTCC, *Bglap*‐reverse: CCATAGATGCGTTTGTAGGCGG, *Hmox1*‐forward: CACTCTGGAGATGACACCTGAG, *Hmox1*‐reverse: GTGTTCCTCTGTCAGCATCACC, *Nrf2*‐forward: CAGCATAGAGCAGGACATGGAG, *Nrf2*‐reverse: GAACAGCGGTAGTATCAGCCAG; *Gapdh*‐forward: CATCACTGCCACCCAGAAGACTG, *Gapdh*‐reverse: ATGCCAGTGAGCTTCCCGTTCAG, *Il1α*‐forward: CGAAGACTACAGTTCTGCCATT, *Il1α*‐reverse: GACGTTTCAGAGGTTCTCAGAG, *Il1β*‐forward: TGGACCTTCCAGGATGAGGACA, *Il1β*‐reverse: GTTCATCTCGGAGCCTGTAGTG.

### 5.13 Dual‐Luciferase Reporter Gene Assay

PGL4.10‐Hmox1 promoter (WT)‐LUC, PGL4.10‐Hmox1 promoter (Mutant)‐LUC, and control PGL4.10 plasmid were provided by HeYuan Biotech, Shanghai. pCGN‐CMV‐Bmal1‐HA plasmid and pRL‐CMV‐Renilla plasmid were provided by Ying Xu's lab at the Cambridge‐Soochow Genomic Research Group, Soochow University. 293 T cells were cultured in DMEM medium containing 10% FBS and 1% PS. When the cell density reached about 70%, plasmid transfection was performed using Hieff Trans Liposomal Transfection Reagents (Yeason, 40802ES). After 48 h, a Dual Luciferase Reporter Assay Kit (Vazyme, DL101) was used for fluorescence signal detection with BioTek Synergy H1.

### 5.14 C2C12 Myogenesis Differentiation, Adenovirus Infection, and Drug Treatment

C2C12 cells were purchased from Procell and cultured in DMEM medium containing 10% FBS and 1% PS. After reaching 80% confluence, the medium was replaced with DMEM differentiation medium containing 2% horse serum and 1% PS, and the medium was changed daily. One week later, clear myotube formation could be observed.

For adenovirus infection, add 300 μL of DMEM growth medium containing 2 × 10^6^ pfu/mL adenovirus to the myotubes in 24‐well plates, and after 6 h, supplement with 200 μL of differentiation medium. Continue culturing in differentiation medium for 4–5 days to allow full adenovirus expression.

### 5.15 C2C12 Myotube ROS Detection

Cells were washed once with serum‐free DMEM and subsequently incubated with serum‐free DMEM containing 10 μM 2′,7′‐dichlorodihydrofluorescein diacetate (DCFH‐DA; Elabscience, E‐BC‐K138‐F) at 37°C for 2 h in a humidified 5% CO_2_ atmosphere. Following incubation, intracellular reactive oxygen species (ROS) levels were quantified through fluorescence intensity measurement using a microplate reader (excitation/emission: 488/525 nm).

### 5.16 Serum Inflammatory Cytokine Chip

Inflammatory cytokine profiling was performed using the Mouse Inflammation Array GS1 Antibody Chip (Raybiotech, GSM‐INF‐1‐1) according to the manufacturer's protocol, with subsequent data analysis conducted by Shanghai Yujin Biotech Co. Ltd.

### 5.17 ELISA

Culture supernatants from differentiated myotubes or serum samples collected from experimental mice were analyzed for IL‐1α and IL‐13 concentrations using commercially available enzyme‐linked immunosorbent assay (ELISA) kits (Mouse IL‐1α ELISA Kit, Elabscience, E‐EL‐M0037c; Mouse IL‐13 ELISA Kit, Elabscience, E‐EL‐M0044c; Mouse IL‐1β ELISA Kit, ELK biotech, ELK1271) according to the manufacturer's standardized protocols. All samples were assayed in technical duplicates, and cytokine concentrations were determined against a standard curve generated from recombinant protein standards provided with each kit.

### 5.18 Primary Skeletal Muscle Cell Isolation

Male C57BL/6 mice (postnatal 1–2 months) were euthanized via cervical dislocation. Following surface sterilization with 75% ethanol, gastrocnemius muscles were dissected, cleared of adipose/connective tissue, and minced into ≈0.1‐mm^3^ fragments. Tissue fragments were digested in DMEM containing 1 mg/mL each of collagenase types I, II, IV, and dispase (37°C, 60 min). The resultant suspension was filtered through a 70‐μm mesh, centrifuged, washed with DMEM, and cultured in DMEM supplemented with 10% FBS under standard conditions (37°C, 5% CO_2_). Cell purity was assessed by α‐SMA immunofluorescence.

### 5.19 Oil Red Staining

Fresh gastrocnemius tissues were fixed in 4% paraformaldehyde for ≥ 24 h. Tissues were then dehydrated sequentially in 15% and 30% sucrose solutions at 4°C until settled. Dehydrated tissues were embedded in OCT compound and rapidly frozen. Cryosections (8–10 μm thick) were cut and collected onto glass slides. Cryosections were air‐dried at room temperature for 10 min and were incubated in Oil Red O working solution for 10 min for sections and washed. Sections were differentiated in 75% ethanol (2 s), rinsed in water (1 min), counterstained with Harris hematoxylin (1 min), differentiated in 1% acid ethanol, blued in ammonia water, rinsed, and mounted with glycerol gelatin. Images were acquired and fibrosis differences were analyzed using ImageJ software.

### 5.20 Picro Sirius Red Staining

Paraffin‐embedded muscle tissue sections were deparaffinized and rehydrated through sequential immersion in xylene I (12 min), xylene II (12 min), absolute ethanol I (6 min), 95% ethanol (6 min), and 85% ethanol (6 min), followed by a 2‐min rinse in tap water. Sections were then stained in saturated picric acid Sirius red solution for 8 min and rinsed briefly in absolute ethanol. After air‐drying in a 60°C oven, sections were cleared in xylene for 5 min and mounted with neutral balsam. Images were acquired, and fibrosis differences were analyzed using ImageJ software.

### 5.21 Co‐Immunoprecipitation

The plasmids pCMV‐T7‐MCS‐3xFlag (MiaoLing, P39495), pCMV‐Bmal1(mouse)‐3xMyc (MiaoLing, P85848), and pCMV‐Nrf2‐3xFlag (MiaoLing, P92972) were obtained from Miaoling plasmid platform. Forty‐eight hours posttransfection in 6‐well plates, cells were lysed using RIPA buffer (Epizyme, PC103), and protein supernatants were collected. The supernatants were incubated with Myc primary antibody (Proteintech, 60,003–2‐Ig) at a 1:100 dilution overnight at 4°C, followed by the addition of 25 μL of Protein A/G beads (MCE, HY‐K0202) for an additional 2 h. The beads were washed five times with lysis buffer, resuspended in loading buffer, and denatured at 95°C for 5 min prior to Western blotting. The expression of BMAL1, NRF2, and GAPDH was detected using anti‐Flag (Sigma F1804, 1:1000), anti‐Myc (CST 2278S, 1:1000), and anti‐GAPDH (Proteintech 60,004–1‐Ig, 1:3000) antibodies, respectively.

### 5.22 Single‐Cell Visualization and Cross‐Cell‐Type Differential Analysis

Human and mouse skeletal muscle single‐cell and single‐nucleus RNA sequencing data were obtained from the publicly available Muscle Aging Cell Atlas (Kedlian et al. 2024) by directly downloading the published h5ad objects, which were imported into R and converted to SingleCellExperiment objects for subsequent analyses. All analyses were performed using the processed data provided by the original authors, including the normalized expression matrix, precomputed dimensionality reduction embeddings, and cell type annotations, with human cell types classified according to annotation_level2 to preserve fine‐grained resolution and mouse cell types retained in their original form without any reclustering or reannotation. Age group definitions (Young vs. Old) were adopted strictly from the original metadata fields (Age_bin or Age_group), and no reclassification or merging of age intervals was applied. UMAP visualizations were generated directly from precalculated coordinates and colored by either cell type annotation or age group. Gene expression values were extracted from the normalized expression matrix. To evaluate age‐related differential expression within each cell type, a two‐sided Wilcoxon rank‐sum test was performed between the young and old groups, and *p*‐values were adjusted using the Benjamini–Hochberg method to control the false discovery rate (FDR). For specific target genes, volcano plots were constructed with log2 fold change on the *x*‐axis and –log10(FDR) on the *y*‐axis. In these plots, each point represents one cell type; point size reflects the proportion of cells with detectable expression (expression > 0) within that cell type, and point color denotes the mean expression level, with specific visualization thresholds detailed in the corresponding figure legends. Additionally, an independent murine single‐cell RNA sequencing dataset used for supplemental validation was obtained from the Aging Atlas Consortium (2021).

### 5.23 Muscle Fiber Analysis

Mouse gastrocnemius and quadriceps muscles were isolated and fixed in GD fixative (Haoke, HK2013) at room temperature for 24 h. The fixed tissues were then embedded in paraffin and cut into 4‐μm‐thick sections. Following hematoxylin and eosin (H&E) staining, the sections were imaged using a digital whole‐slide pathology scanner (Jiangfeng, KF‐FL‐020). The cross‐sectional area (CSA) of the muscle fibers was subsequently quantified using ImageJ software.

### 5.24 Data Analysis

Statistical analyses were performed using GraphPad Prism 8.0 (GraphPad Software Inc., San Diego, CA, USA). For comparisons between two groups, two‐tailed Student's *t*‐tests were applied, while two‐way analysis of variance (ANOVA) was employed for analyzing differences between curves. The threshold for statistical significance was set at *p* < 0.05.

## Author Contributions

Q.Z., Y.X.: conceptualization, writing – review and editing, funding acquisition. K.H., J.Q., Y.W., Q.Z.: project administration, validation. K.H.: writing – original draft. K.H., Y.W., J.Q., F.Z.: data curation, formal analysis.

## Funding

This work was supported by Natural Science Foundation of Zhejiang Province, ZCLQ24H0701. Quzhou Municipal Science and Technology Bureau, 2023K107. National Natural Science Foundation of China, 82372455.

## Conflicts of Interest

The authors declare no conflicts of interest.

## Supporting information


**Figure S1:** Age‐related musculoskeletal phenotypes in male mice. (A) Representative 3D reconstructed micro‐CT images of the distal femoral trabecular and cortical bone in 2‐month‐old (young) and 18‐month‐old (aged) male mice. (B–I) Quantitative analysis of femoral bone parameters: bone mineral density (BMD), bone volume fraction (BV/TV), trabecular number (Tb.N), trabecular separation (Tb.Sp), trabecular thickness (Tb.Th), cortical bone mineral density (Ct.BMD), cortical thickness (Ct.Th) and cortical area in 2‐month‐old (*n* = 5) and 18‐month‐old (*n* = 5) male mice. (J, K) Representative immunohistochemical staining for P21 and corresponding quantitative analysis in the gastrocnemius muscle of 2‐month‐old (*n* = 3) and 18‐month‐old (*n* = 3) male mice. (L, M) Representative Oil Red O staining (indicating lipid content) and corresponding quantitative analysis in the gastrocnemius muscle of 2‐month‐old (*n* = 3) and 18‐month‐old (*n* = 3) male mice. (N, O) Representative Picrosirius Red staining (indicating collagen deposition) and corresponding quantitative analysis in the gastrocnemius muscle of 2‐month‐old (*n* = 3) and 18‐month‐old (*n* = 3) male mice. Data are presented as mean ± SD. *p < 0.05, ***p < 0.001, ****p < 0.0001, ns, not significant.

**Figure S2:** Temporal muscle‐bone ligand‐receptor interactions. (A) Heatmap depicting muscle‐derived secretory proteins paired with bone‐expressed receptors across ZT points (ZT1, ZT5, ZT9, ZT13, ZT17, ZT21) in young mice. Color intensity represents interaction strength (Mean). (B) Corresponding ligand‐receptor pairing heatmap in aged mice at identical ZT point.

**Figure S3:** UMAP of human muscle and cell type‐specific *Bmal1* expression in mice skeletal muscle. (A) UMAP of human muscle. (B) Comparative *Bmal1* expression (scRNA‐seq) across myofiber and stromal cell populations: EC, endothelial cells; FAPs, fibro‐adipogenic progenitors; Fast IIB, type IIB fast‐twitch myofibers; Fib, fibroblasts; Mac, macrophages; MJ, myotendinous junction cells; NMJ_pre, neuromuscular junction precursor cells; Per, pericytes; SMC, smooth muscle cells. Tendon cells. Aged versus young mice Tendon cells. Aged versus young mice.

**Figure S4:** Muscle mass, muscle fiber size, and *Mstn* expression in mKO mice. (A) Statistical analysis of the ratio of quadriceps and gastrocnemius weight to body weight in 4‐month‐old mKO and control mice (*n* = 4). (B, C) H&E staining images and statistical analysis of the mean cross‐sectional area of gastrocnemius muscle fibers from mKO and control mice (*n* = 4). (D, E) H&E staining images and statistical analysis of the mean cross‐sectional area of quadriceps muscle fibers from mKO and control mice (*n* = 4). (F) Statistical results of *Mstn* FPKM values in the gastrocnemius muscle of mKO and control mice based on RNA‐seq (*n* = 12). Data are presented as mean ± SD. *p < 0.05.

**Figure S5:** Effects of muscle‐specific *Bmal1* deficiency on the femur in 10‐month‐old mice. (A) Representative 3D micro‐CT reconstructions of trabecular bone in the distal femur of 10‐month‐old male mKO and control mice. (B–F) Quantitative micro‐CT analysis of trabecular bone parameters in the distal femur: BMD, BV/TV, Tb.Sp, Tb.N, and Tb.Th (*n* = 5). Data are presented as mean ± SD. *p < 0.05, **p < 0.01.

**Figure S6:** Pathway enrichment and antioxidant gene dysregulation in aged and mKO mice. (A) Wikipathways enrichment of genes losing rhythmicity in aged muscle. (B) Wikipathways enrichment of 270 genes exhibiting concurrent rhythmicity loss and differential expression in mKO mice. (C) Temporal expression profiles (FPKM) of antioxidant genes *Hmox1* and *Nrf2* in gastrocnemius muscle of aged and young mice across ZT1, ZT5, ZT9, ZT13, ZT17, ZT21 (*n* = 3). Multiple *t*‐tests were used to analyze differences in gene expression at different time points. Data presented as mean ± SD. *p < 0.05, **p < 0.01.

**Figure S7:** Verification of the interaction between NRF2 and BMAL1. (A) Publicly available ChIP‐seq data (Cistrome Database) demonstrating binding signals for both NRF2 (in C2C12 cells and macrophages) and BMAL1 (in liver and heart tissues) within the *Hmox1* promoter region. (B) Co‐immunoprecipitation (Co‐IP) assay detecting Flag‐NRF2 expression following the immunoprecipitation of Myc‐BMAL1 in 293 T cells cotransfected with Flag‐Nrf2 and Myc‐Bmal1 plasmids.

**Figure S8:** Muscle Bmal1 KO affects NF‐κB‐related pathways. GO pathway enrichment analysis of DEGs in muscle *Bmal1* KO versus control group (top 10).

**Figure S9:** Inflammatory mediator profiles in muscle‐*Bmal1*‐KO serum. Relative levels of circulating inflammatory mediators in mKO mice versus littermate controls at ZT8 and ZT20 (*n* = 3). Data presented as mean ± SD.

**Figure S10:** Effects of *Bmal1* deficiency on IL‐1α and IL‐1β expression in C2C12 myotubes and skeletal muscle tissue. (A) qPCR analysis of *Il1a* and *Il1b* mRNA expression in C2C12 myotubes following *Bmal1* knockdown via adenovirus (ADV) infection. (B) ELISA quantification of secreted IL‐1β levels in the myotube culture medium. (C, D) Representative Western blots (C) and statistical quantification (D) of BMAL1, IL‐1α, and IL‐1β protein expression in C2C12 myotubes following ADV‐mediated *Bmal1* knockdown. (E, F) Western blot analysis and statistical quantification of IL‐1α and IL‐1β protein expression in the gastrocnemius muscle of muscle‐specific *Bmal1* knockout mice and control mice. Data are presented as mean ± SD. *p < 0.05, **p < 0.01.

**Figure S11:** Effects of TRF on the transcriptome of the gastrocnemius muscle in aged mice. (A) Number of DEGs between the TRF and AL groups at ZT14 and ZT2 (*p* < 0.05 and fold changes ≥ 1.5). (B) KEGG pathway enrichment analysis of DEGs.

## Data Availability

The data that support the findings of this study are openly available in NCBI SRA at https://www.ncbi.nlm.nih.gov/bioproject/PRJNA1164196, reference number PRJNA1164196.
