# Supplementary figures and images for: Aging‐Related Muscle Bmal1 Decline Contributes to Bone Loss in Mice via Enhancing IL‐1α–Mediated Osteoclastogenesis
Source: Aging Cell. 2026 Jun 8;25(6):e70582. doi: 10.1111/acel.70582 (PMC13246325; doi:10.1111/acel.70582)

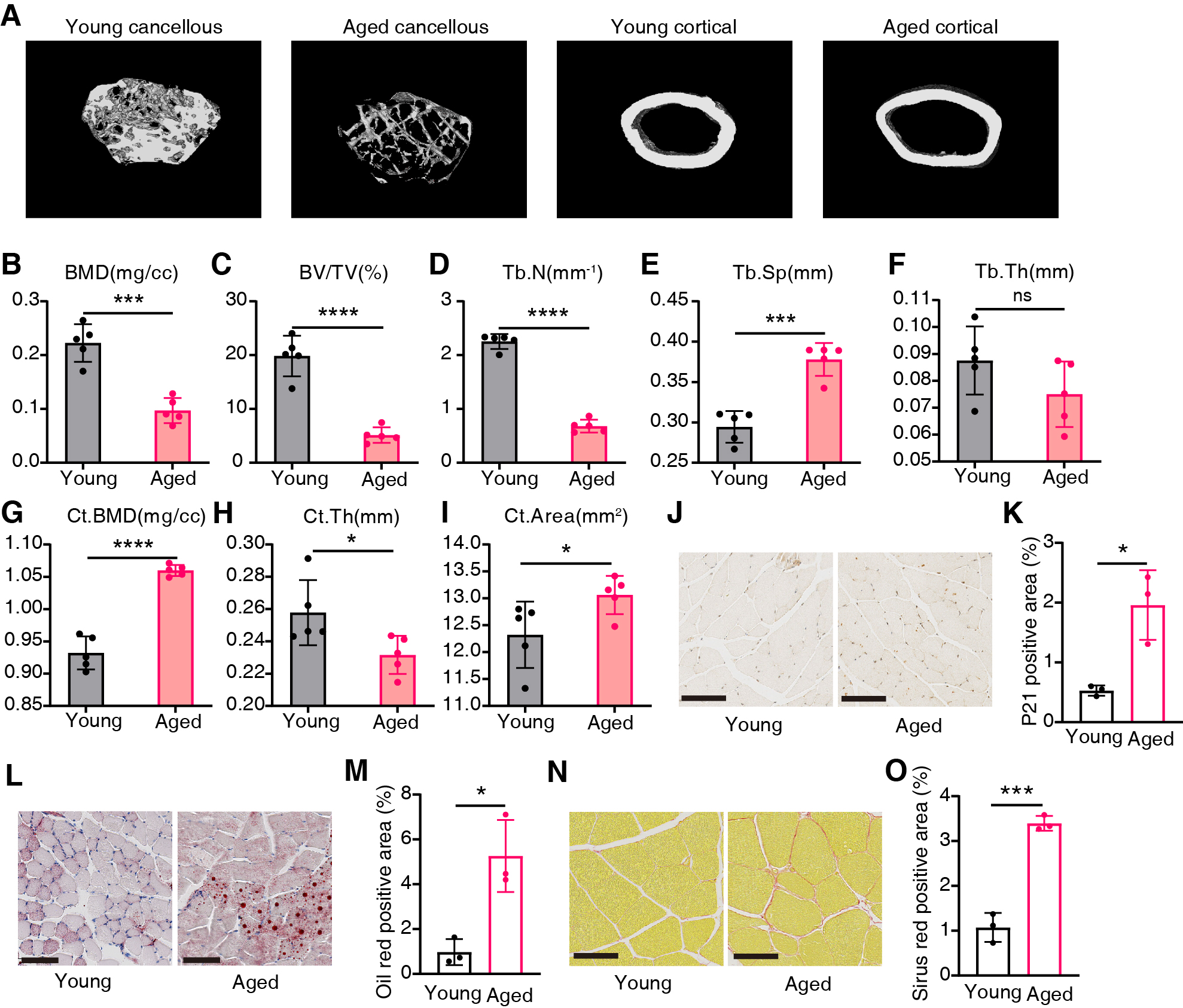

Supplement: Supplementary file 1 — Figure S1: Age‐related musculoskeletal phenotypes in male mice. (A) Representative 3D reconstructed micro‐CT images of the distal femoral trabecular and cortical bone in 2‐month‐old (young) and 18‐month‐old (aged) male mice. (B–I) Quantitative analysis of femoral bone parameters: bone mineral density (BMD), bone volume fraction (BV/TV), trabecular number (Tb.N), trabecular separation (Tb.Sp), trabecular thickness (Tb.Th), cortical bone mineral density (Ct.BMD), cortical thickness (Ct.Th) and cortical area in 2‐month‐old (n = 5) and 18‐month‐old (n = 5) male mice. (J, K) Representative immunohistochemical staining for P21 and corresponding quantitative analysis in the gastrocnemius muscle of 2‐month‐old (n = 3) and 18‐month‐old (n = 3) male mice. (L, M) Representative Oil Red O staining (indicating lipid content) and corresponding quantitative analysis in the gastrocnemius muscle of 2‐month‐old (n = 3) and 18‐month‐old (n = 3) male mice. (N, O) Representative Picrosirius Red staining (indicating collagen deposition) and corresponding quantitative analysis in the gastrocnemius muscle of 2‐month‐old (n = 3) and 18‐month‐old (n = 3) male mice. Data are presented as mean ± SD. *p < 0.05, ***p < 0.001, ****p < 0.0001, ns, not significant. [file ACEL-25-e70582-s001.jpg]

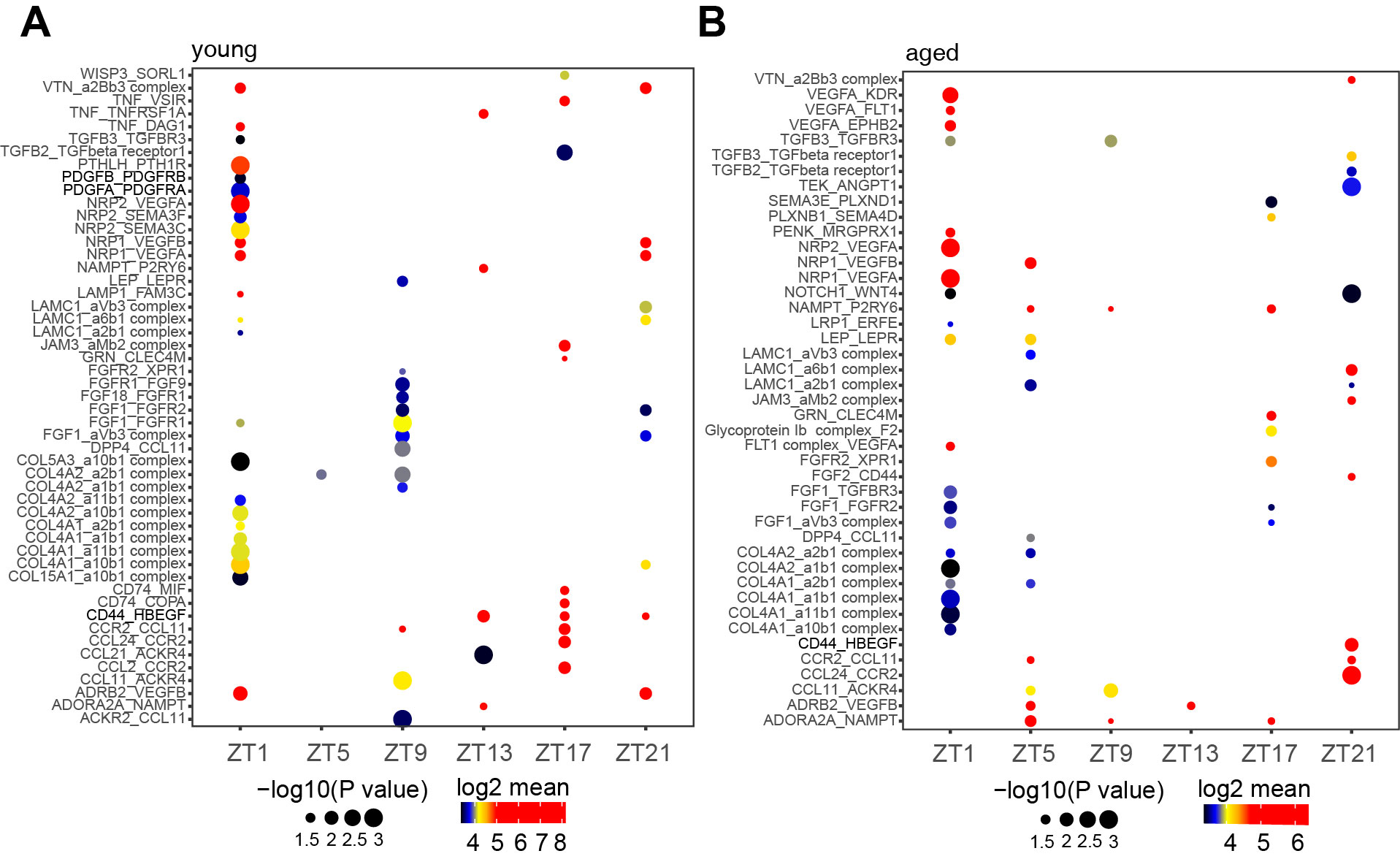

Supplement: Supplementary file 2 — Figure S2: Temporal muscle‐bone ligand‐receptor interactions. (A) Heatmap depicting muscle‐derived secretory proteins paired with bone‐expressed receptors across ZT points (ZT1, ZT5, ZT9, ZT13, ZT17, ZT21) in young mice. Color intensity represents interaction strength (Mean). (B) Corresponding ligand‐receptor pairing heatmap in aged mice at identical ZT point. [file ACEL-25-e70582-s008.jpg]

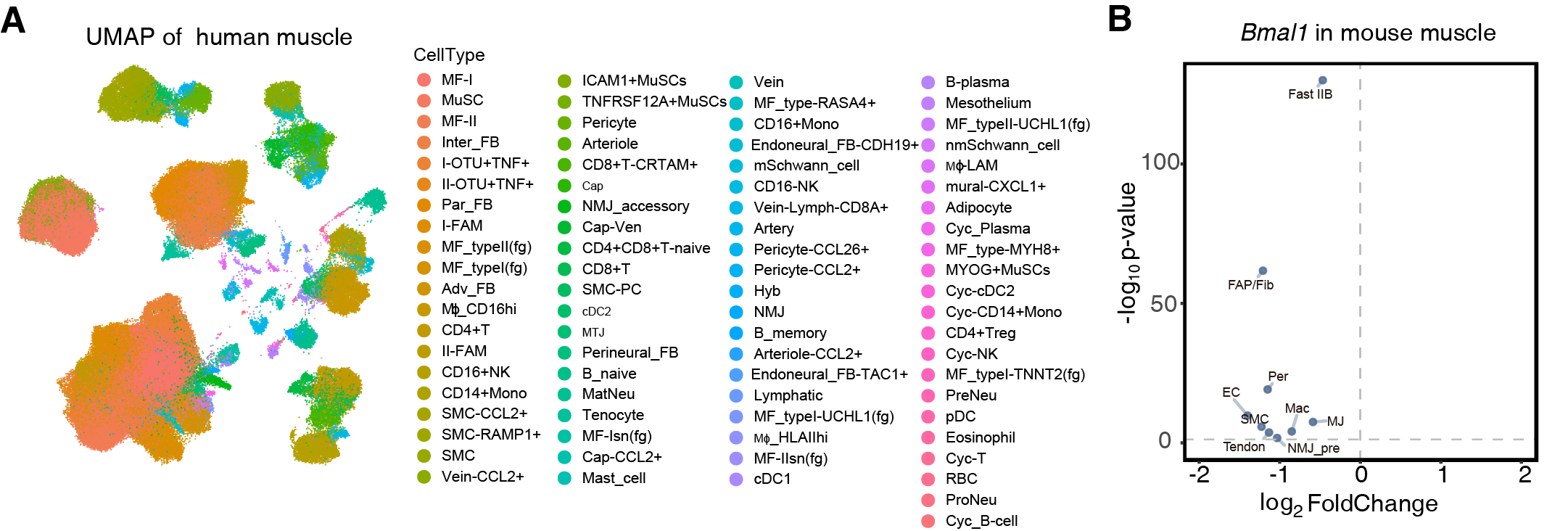

Supplement: Supplementary file 3 — Figure S3: UMAP of human muscle and cell type‐specific Bmal1 expression in mice skeletal muscle. (A) UMAP of human muscle. (B) Comparative Bmal1 expression (scRNA‐seq) across myofiber and stromal cell populations: EC, endothelial cells; FAPs, fibro‐adipogenic progenitors; Fast IIB, type IIB fast‐twitch myofibers; Fib, fibroblasts; Mac, macrophages; MJ, myotendinous junction cells; NMJ_pre, neuromuscular junction precursor cells; Per, pericytes; SMC, smooth muscle cells. Tendon cells. Aged versus young mice Tendon cells. Aged versus young mice. [file ACEL-25-e70582-s004.jpg]

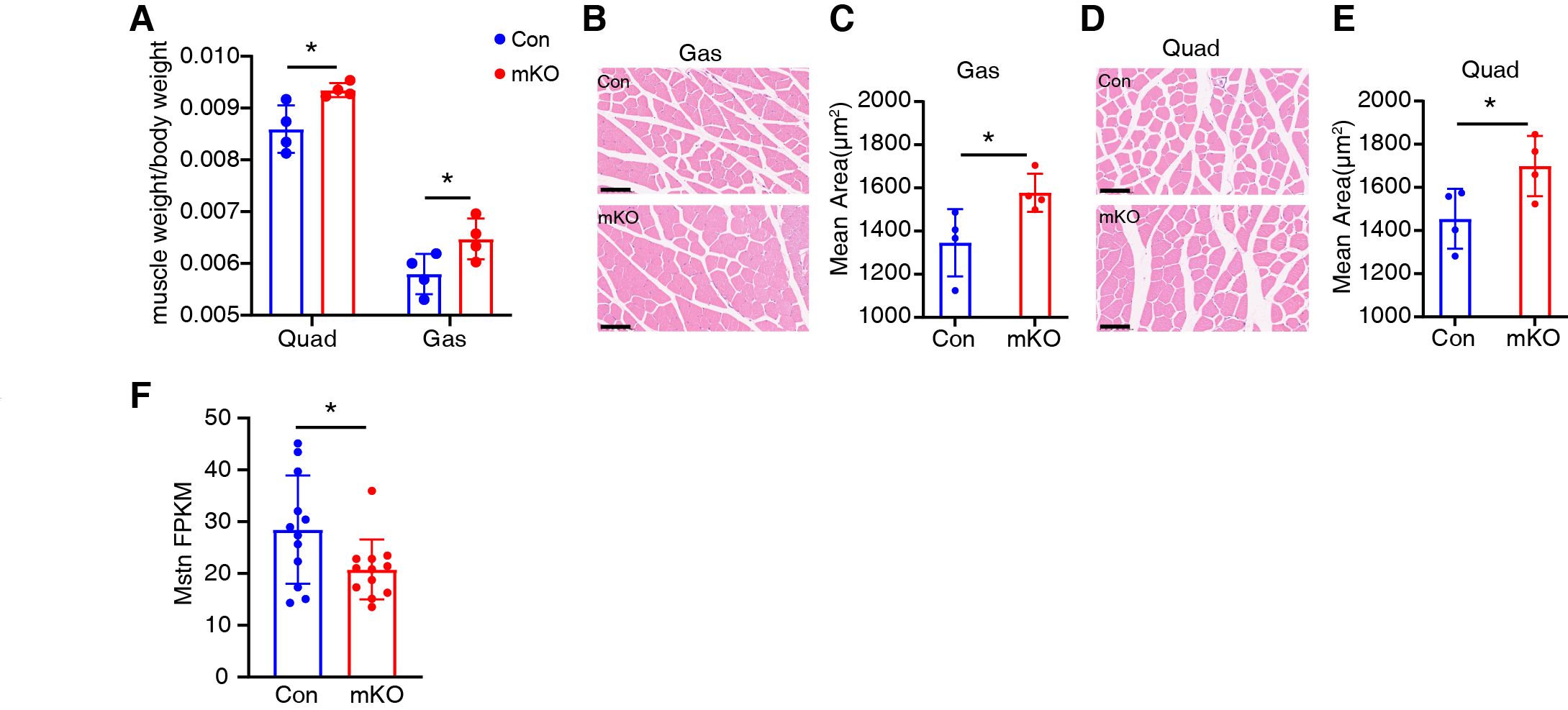

Supplement: Supplementary file 4 — Figure S4: Muscle mass, muscle fiber size, and Mstn expression in mKO mice. (A) Statistical analysis of the ratio of quadriceps and gastrocnemius weight to body weight in 4‐month‐old mKO and control mice (n = 4). (B, C) H&E staining images and statistical analysis of the mean cross‐sectional area of gastrocnemius muscle fibers from mKO and control mice (n = 4). (D, E) H&E staining images and statistical analysis of the mean cross‐sectional area of quadriceps muscle fibers from mKO and control mice (n = 4). (F) Statistical results of Mstn FPKM values in the gastrocnemius muscle of mKO and control mice based on RNA‐seq (n = 12). Data are presented as mean ± SD. *p < 0.05. [file ACEL-25-e70582-s003.jpg]

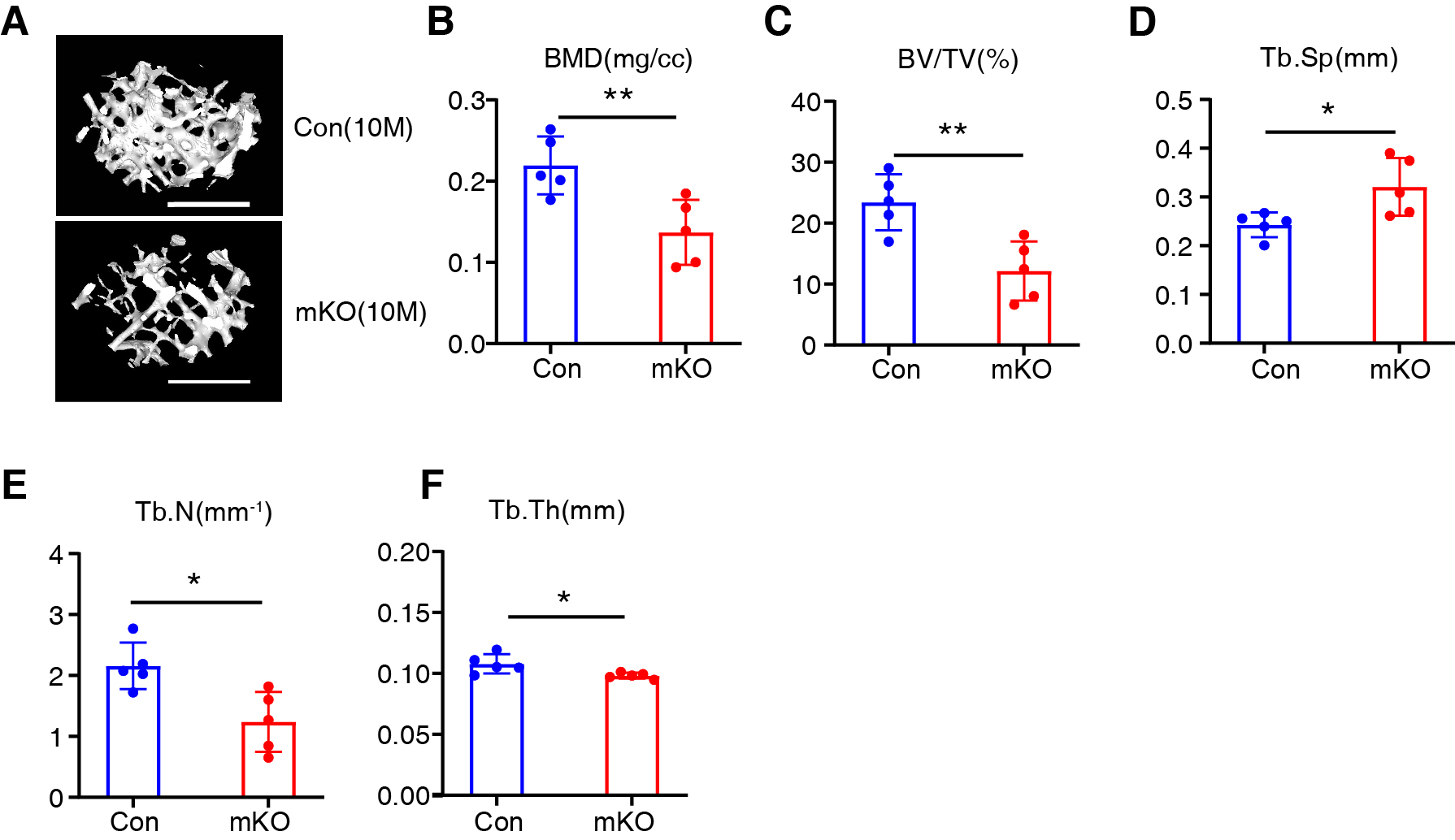

Supplement: Supplementary file 5 — Figure S5: Effects of muscle‐specific Bmal1 deficiency on the femur in 10‐month‐old mice. (A) Representative 3D micro‐CT reconstructions of trabecular bone in the distal femur of 10‐month‐old male mKO and control mice. (B–F) Quantitative micro‐CT analysis of trabecular bone parameters in the distal femur: BMD, BV/TV, Tb.Sp, Tb.N, and Tb.Th (n = 5). Data are presented as mean ± SD. *p < 0.05, **p < 0.01. [file ACEL-25-e70582-s010.jpg]

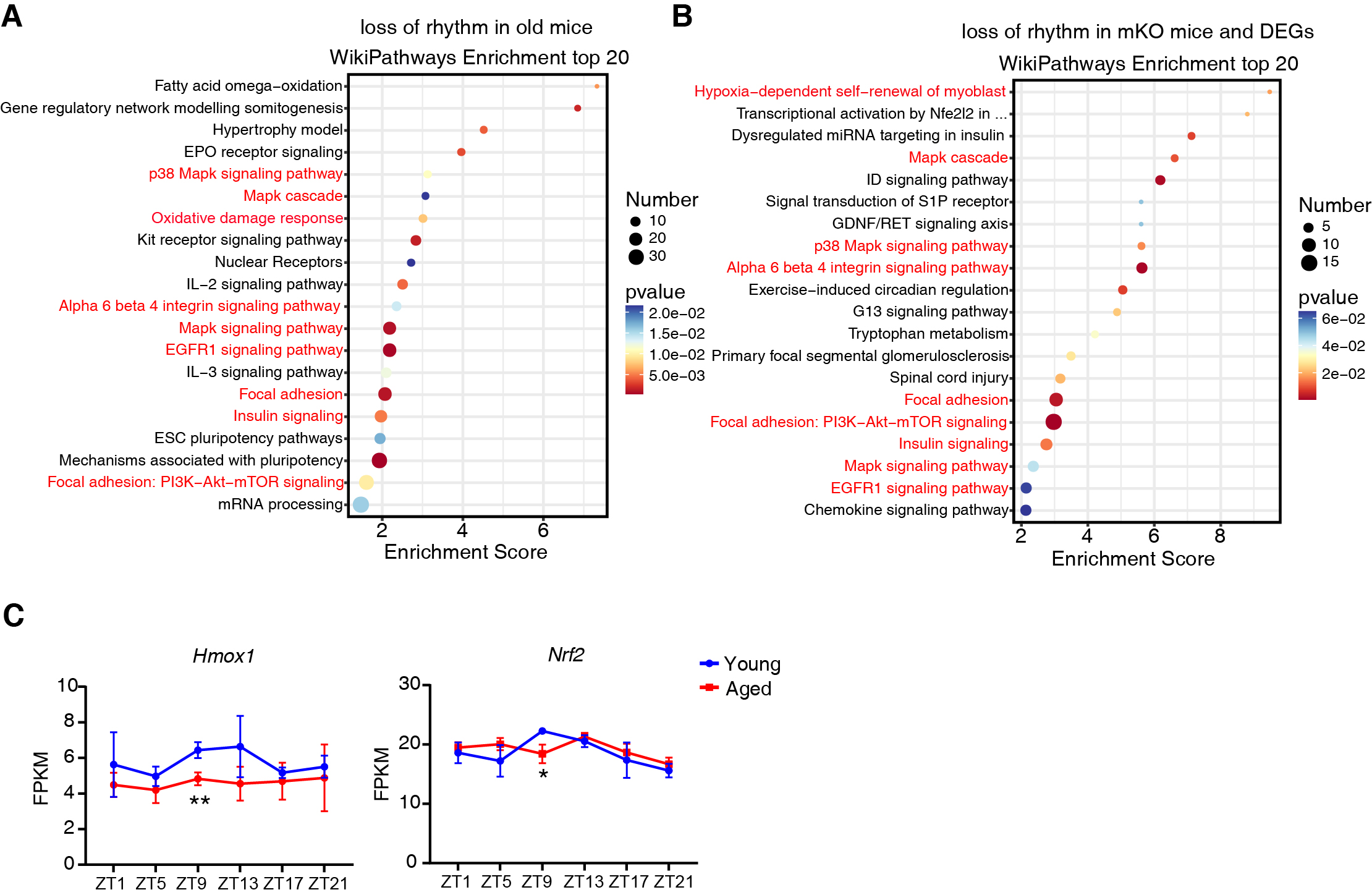

Supplement: Supplementary file 6 — Figure S6: Pathway enrichment and antioxidant gene dysregulation in aged and mKO mice. (A) Wikipathways enrichment of genes losing rhythmicity in aged muscle. (B) Wikipathways enrichment of 270 genes exhibiting concurrent rhythmicity loss and differential expression in mKO mice. (C) Temporal expression profiles (FPKM) of antioxidant genes Hmox1 and Nrf2 in gastrocnemius muscle of aged and young mice across ZT1, ZT5, ZT9, ZT13, ZT17, ZT21 (n = 3). Multiple t‐tests were used to analyze differences in gene expression at different time points. Data presented as mean ± SD. *p < 0.05, **p < 0.01. [file ACEL-25-e70582-s011.jpg]

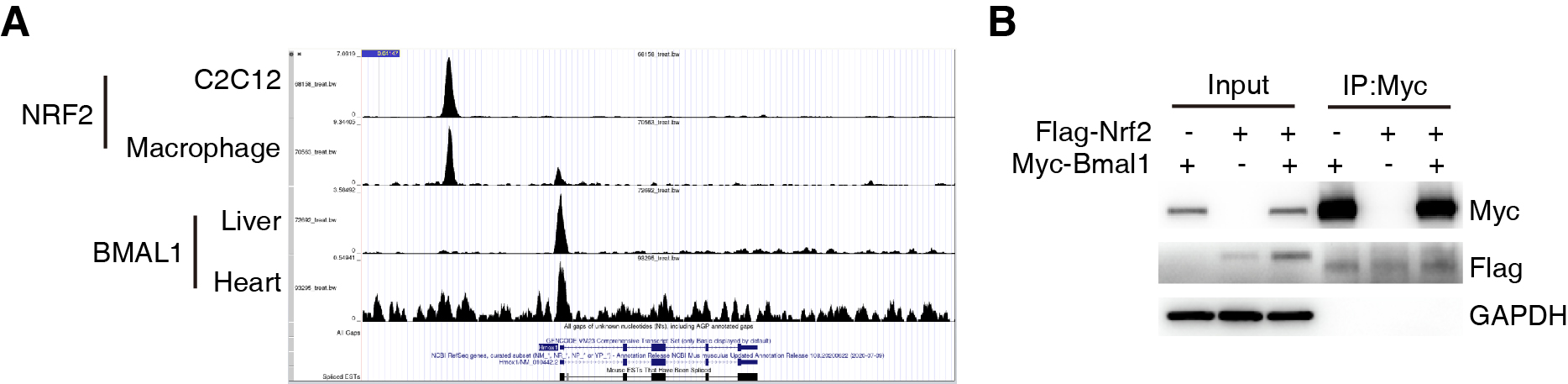

Supplement: Supplementary file 7 — Figure S7: Verification of the interaction between NRF2 and BMAL1. (A) Publicly available ChIP‐seq data (Cistrome Database) demonstrating binding signals for both NRF2 (in C2C12 cells and macrophages) and BMAL1 (in liver and heart tissues) within the Hmox1 promoter region. (B) Co‐immunoprecipitation (Co‐IP) assay detecting Flag‐NRF2 expression following the immunoprecipitation of Myc‐BMAL1 in 293 T cells cotransfected with Flag‐Nrf2 and Myc‐Bmal1 plasmids. [file ACEL-25-e70582-s009.jpg]

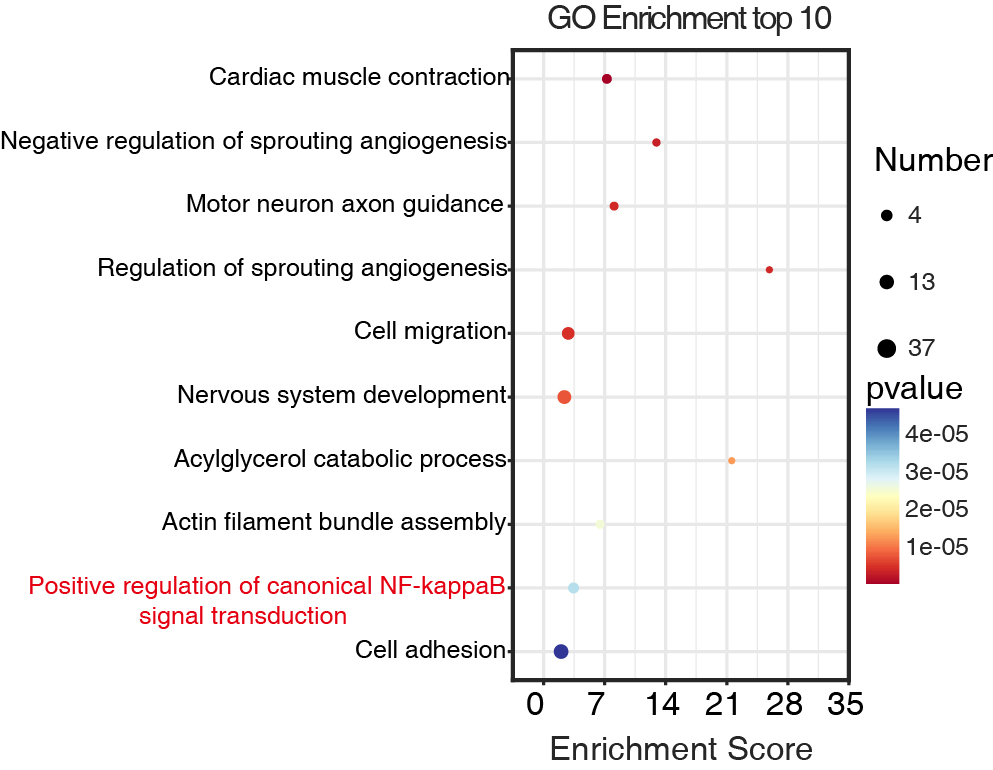

Supplement: Supplementary file 8 — Figure S8: Muscle Bmal1 KO affects NF‐κB‐related pathways. GO pathway enrichment analysis of DEGs in muscle Bmal1 KO versus control group (top 10). [file ACEL-25-e70582-s007.jpg]

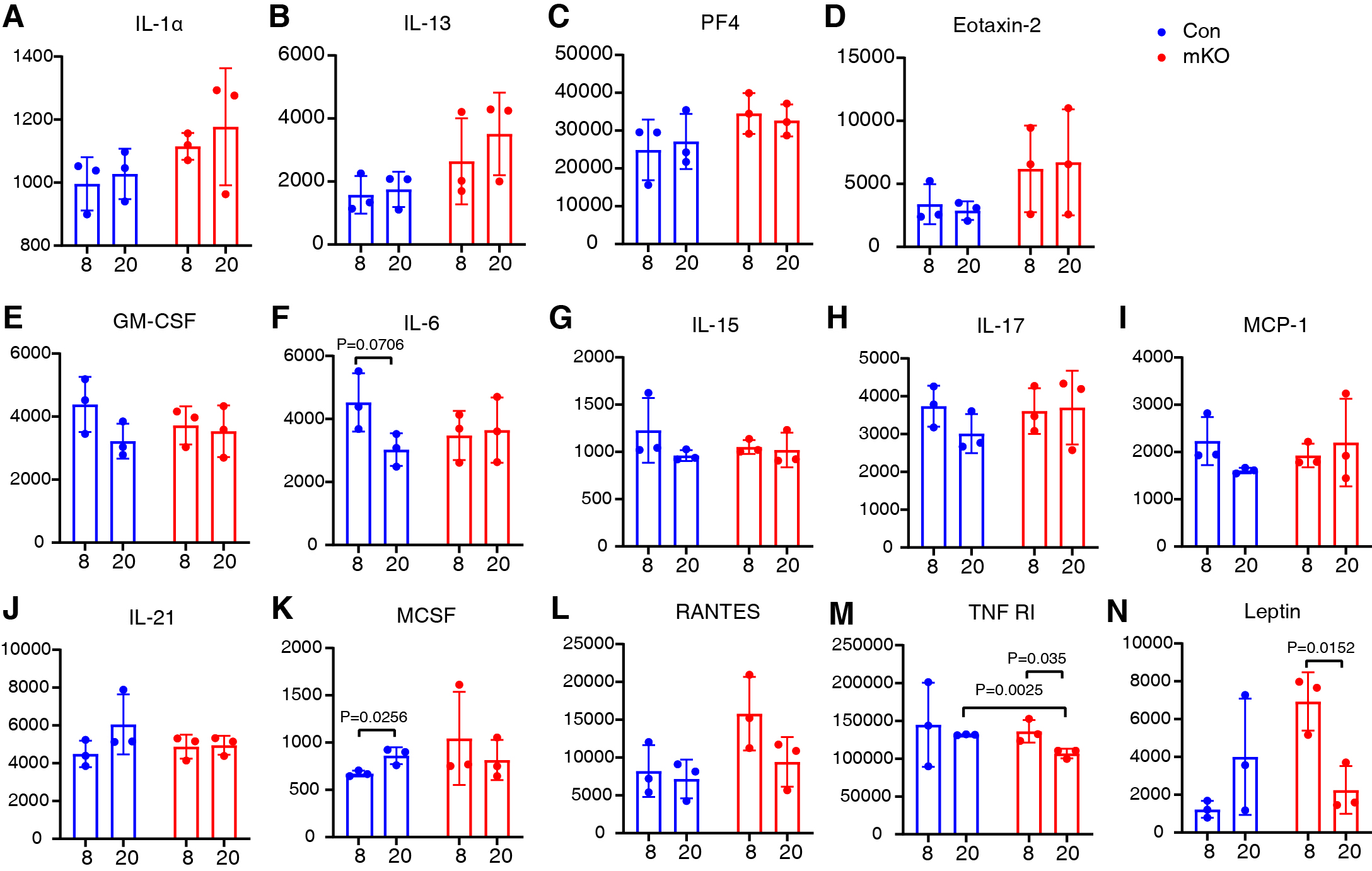

Supplement: Supplementary file 9 — Figure S9: Inflammatory mediator profiles in muscle‐Bmal1‐KO serum. Relative levels of circulating inflammatory mediators in mKO mice versus littermate controls at ZT8 and ZT20 (n = 3). Data presented as mean ± SD. [file ACEL-25-e70582-s002.jpg]

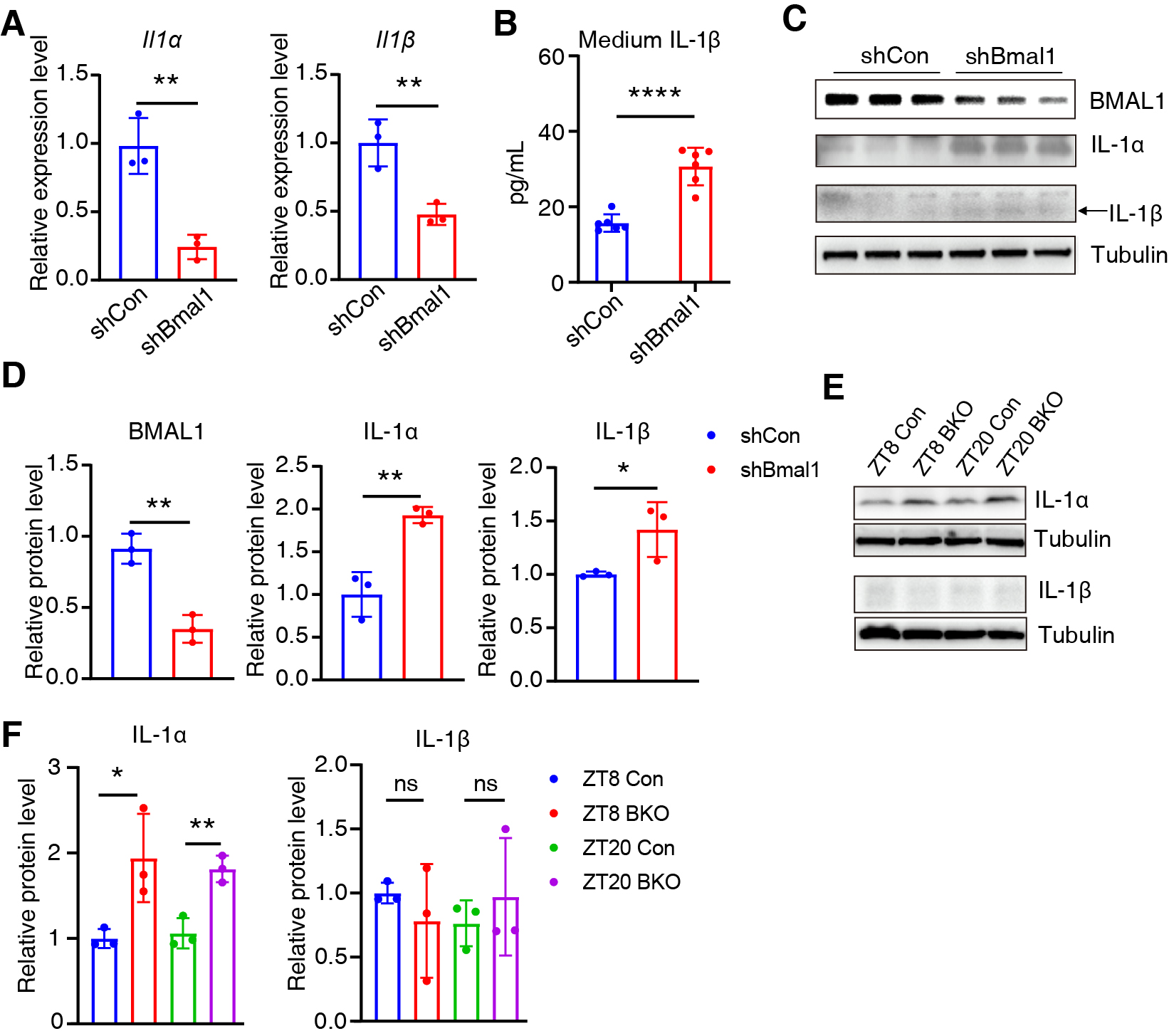

Supplement: Supplementary file 10 — Figure S10: Effects of Bmal1 deficiency on IL‐1α and IL‐1β expression in C2C12 myotubes and skeletal muscle tissue. (A) qPCR analysis of Il1a and Il1b mRNA expression in C2C12 myotubes following Bmal1 knockdown via adenovirus (ADV) infection. (B) ELISA quantification of secreted IL‐1β levels in the myotube culture medium. (C, D) Representative Western blots (C) and statistical quantification (D) of BMAL1, IL‐1α, and IL‐1β protein expression in C2C12 myotubes following ADV‐mediated Bmal1 knockdown. (E, F) Western blot analysis and statistical quantification of IL‐1α and IL‐1β protein expression in the gastrocnemius muscle of muscle‐specific Bmal1 knockout mice and control mice. Data are presented as mean ± SD. *p < 0.05, **p < 0.01. [file ACEL-25-e70582-s006.jpg]

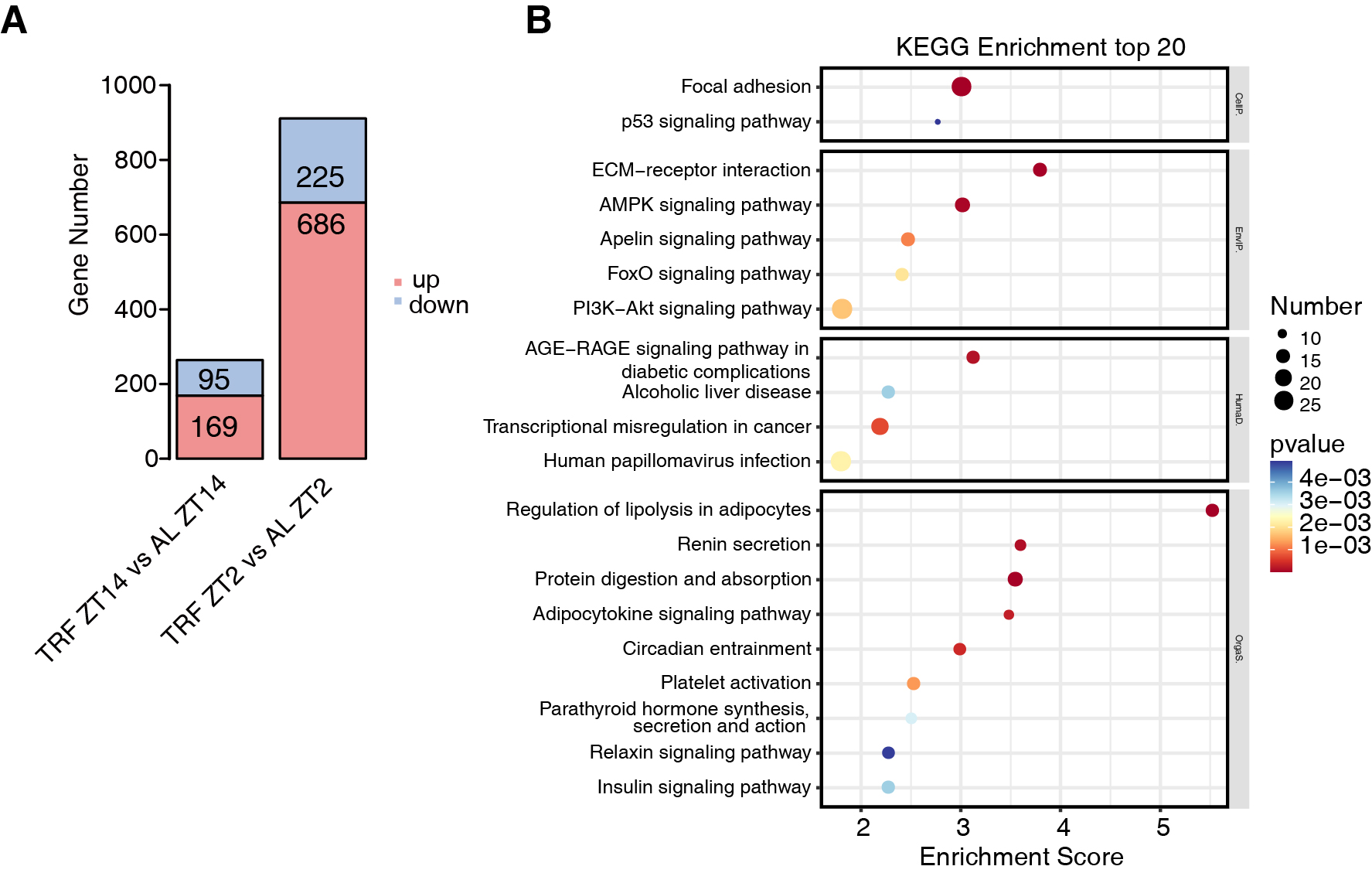

Supplement: Supplementary file 11 — Figure S11: Effects of TRF on the transcriptome of the gastrocnemius muscle in aged mice. (A) Number of DEGs between the TRF and AL groups at ZT14 and ZT2 (p < 0.05 and fold changes ≥ 1.5). (B) KEGG pathway enrichment analysis of DEGs. [file ACEL-25-e70582-s005.jpg]
